# Regenerating Immunotolerance in Multiple Sclerosis with Autologous Hematopoietic Stem Cell Transplant

**DOI:** 10.3389/fimmu.2018.00410

**Published:** 2018-03-12

**Authors:** Jennifer C. Massey, Ian J. Sutton, David D. F. Ma, John J. Moore

**Affiliations:** ^1^Haematology and Bone Marrow Transplantation, St Vincent’s Hospital Sydney, Darlinghurst, NSW, Australia; ^2^Neurology, St Vincent’s Hospital Sydney, Darlinghurst, NSW, Australia; ^3^Centre for Applied Medical Research, St Vincent’s Hospital Sydney, Darlinghurst, NSW, Australia; ^4^St Vincent’s Clinical School, University of New South Wales, Sydney, NSW, Australia

**Keywords:** multiple sclerosis, autologous hematopoietic stem cell transplantation, T cell receptor repertoire, immune tolerance, lymphopenia-induced proliferation, alemtuzumab, cladribine

## Abstract

Multiple sclerosis (MS) is an inflammatory disorder of the central nervous system where evidence implicates an aberrant adaptive immune response in the accrual of neurological disability. The inflammatory phase of the disease responds to immunomodulation to varying degrees of efficacy; however, no therapy has been proven to arrest progression of disability. Recently, more intensive therapies, including immunoablation with autologous hematopoietic stem cell transplantation (AHSCT), have been offered as a treatment option to retard inflammatory disease, prior to patients becoming irreversibly disabled. Empirical clinical observations support the notion that the immune reconstitution (IR) that occurs following AHSCT is associated with a sustained therapeutic benefit; however, neither the pathogenesis of MS nor the mechanism by which AHSCT results in a therapeutic benefit has been clearly delineated. Although the antigenic target of the aberrant immune response in MS is not defined, accumulated data suggest that IR following AHSCT results in an immunotolerant state through deletion of pathogenic clones by a combination of direct ablation and induction of a lymphopenic state driving replicative senescence and clonal attrition. Restoration of immunoregulation is evidenced by changes in regulatory T cell populations following AHSCT and normalization of genetic signatures of immune homeostasis. Furthermore, some evidence exists that AHSCT may induce a rebooting of thymic function and regeneration of a diversified naïve T cell repertoire equipped to appropriately modulate the immune system in response to future antigenic challenge. In this review, we discuss the immunological mechanisms of IR therapies, focusing on AHSCT, as a means of recalibrating the dysfunctional immune response observed in MS.

## Introduction

Multiple sclerosis (MS) is an inflammatory condition of the central nervous system (CNS) that affects over 2.3 million people worldwide and is the commonest cause of non-traumatic neurological disability in young adults, with a median age of onset of 34 years (1). In the majority of patients, the disease follows a relapsing and remitting course [relapsing-remitting multiple sclerosis (RRMS)], with up to 80% of individuals entering a secondary progressive (SPMS) phase of the disease associated with gradual neurological decline. While the transition from RRMS to SPMS remains difficult to define, it typically occurs between 10 and 20 years from first MS symptoms (2). The early phase of the disease is marked by episodic inflammation. Once patients have reached the progressive disease phenotype, degeneration becomes the predominant feature of the disease and fails to respond to immunotherapy. Longitudinal studies have demonstrated the median time from disease onset to a requirement of a cane to walk 100 m is 27.9 years (3) and a recent meta-analysis reported a 2.8-fold increase in mortality ratio in MS, predominately in patients with Expanded Disability Status Scale (EDSS) scores >6, as a consequence of bulbar or respiratory muscle dysfunction (4). Suppression of inflammatory activity is the cornerstone of MS treatment and the introduction of disease-modifying therapy (DMT) for MS in the mid-1990s demonstrated that immune modulation could reduce the rate of clinical relapses and accompanying magnetic resonance imaging (MRI) changes of inflammation (5), in turn leading to a reduction in the rate of accumulation of disability. It is well established that early initiation of immunotherapy following first presentation of RRMS is associated with improved long-term outcomes (6). The introduction of immunosuppressive strategies in the management of MS has resulted in improved treatment efficacy (7), although it is becoming increasingly clear that the requirement for long-term maintenance dosing is associated with a small but significant risk of opportunistic infection, most notably progressive multifocal leukoencephalopathy, and possibly malignancy (8–11). Immune reconstitution (IR) therapies encompass a heterogeneous group of pulsed lympho- or myeloablative treatments designed to transiently or permanently induce an immune “reset.” Over the last decade, there has been increasing interest in high-efficacy IR therapies, including chemotherapeutics, monoclonal antibodies such as alemtuzumab, and autologous hematopoietic stem cell transplantation (AHSCT) in autoimmune disease (AID), represented by a surge in clinical trials (12–15) and reviews in the literature (15–19). This has been underpinned by an understanding that appropriate application of IR therapies can induce long-term disease remission, potentially avoiding the need for further treatment.

Immunoablation followed by AHSCT has been utilized as a therapeutic intervention in aggressive AID over the past two decades, following proof of principle experiments using animal models of disease (20–26) and reports of co-incidental improvement of AID symptoms in patients undergoing transplantation for hematological malignancies (27, 28). Therapeutic trials of total body irradiation (TBI) or cyclophosphamide and busulfan (29) with allogeneic bone marrow transplantation (BMT) in rats with experimental allergic encephalomyelitis (EAE), a rodent model of CNS inflammation, and syngeneic grafting experiments (pseudoautologuous HSCT) showed that transplantation can induce disease remission, prevent relapses and enhance recovery from paresis (30, 31). Given the preferable safety record of autologous transplantation in comparison to allografting, particularly in the non-malignant setting, autologous HSCT was deemed acceptable for clinical trials in AID by the mid-1990s. The pivotal report of feasibility of AHSCT in MS was published in 1997 (32), following a cohort of 15 patients with progressive disease who underwent transplantation from 1995. Since then, over 25 phase I and II clinical trials have been published, expanding our understanding of the role of AHSCT in MS (32–42). It is increasingly recognized that AHSCT is more efficacious and associates with a lower mortality rate when applied earlier in the inflammatory phase of the disease than later in the progressive phase (42), at which time there is minimal residual inflammation and significant disability increases the risks of the procedure. However, immunoablation strategies are not without complication and associate with infertility and a dramatic increase in the short-term rate of cerebral atrophy, which is a correlate of acquired neurological disability. It is anticipated that a number of randomized controlled trials planned for the near future (18, 43) will establish the role of AHSCT in the management of treatment-refractory and aggressive MS. Continued investigation into the immunobiological changes occurring following application of AHSCT in MS is needed to facilitate the development of IR therapies with a lower treatment associated mortality and morbidity risk.

## MS Pathogenesis

Multiple lines of evidence have established that a significant proportion of the disability acquired in patients with MS arises as a result of inflammation mediated by an antigen-specific immune response. Genetic studies have consistently confirmed that the MHC class II HLA-DRB1 gene, HLA-DRB1*15:01 is the most important risk factor for the development of MS (44–48), increasing the risk of disease threefold (49). Molecular studies have identified shared clonal populations of T cells between the peripheral blood, cerebrospinal fluid (CSF) and CNS of MS patients (50, 51) and oligoclonal immunoglobulins (Igs) are identified in the CSF, but not serum, of >95% of MS patients (52). T cell receptor (TCR) analysis of MS lesions post mortem has identified clones across anatomically distinct regions (53) with “silent” nucleotide exchanges (different nucleotides coding for the same amino acid) within the V-CDR3-J region, supporting the concept of T cell recruitment to a common antigenic driver of disease. Additionally, genome-wide association studies have identified more than 150 single-nucleotide polymorphisms (SNPs), common genetic variants (54) linked with MS. The majority of these encode for cytokines, cytokine receptors, transcription factors, and costimulatory molecules of the adaptive immune system, indicating that the immune phenotype is critical to disease susceptibility.

The concept of MS as a prototypical AID, where self-reactive lymphocytes induce inflammation in response to myelin or myelin cross-reactive epitopes (55) is based on EAE disease models (56–58) and supported by the presence of myelin-reactive Th1 and Th17 cells (59–61) and their cytokines in the CNS and CSF of MS patients (55). However, while myelin antigens are attractive candidates for an autoreactive immune response, it is accepted that the antigenic target(s) of the clonally expanded lymphocytes in MS remains unknown at present. Inadequacies in the widely accepted theory of MS resulting from CNS-specific autoreactive lymphocytes has been exemplified by historical studies in to the association between infectious agents and MS (62) and recent work investigating the role of human endogenous retroviruses in triggering a pathogenic immune response within the CNS (63, 64). EAE has successfully served as a model of CNS inflammation for development of certain disease-modifying therapies (58), but while widely cited as “an animal model of MS” EAE should not be viewed as such, since induction of EAE results in widespread inflammatory change that lacks specificity for oligodendrocyte injury and the perivascular and sub-pial distribution of the neuropathological changes observed in EAE (65) are quite distinct from the pathology that is observed in MS (66).

**Figure 1:**
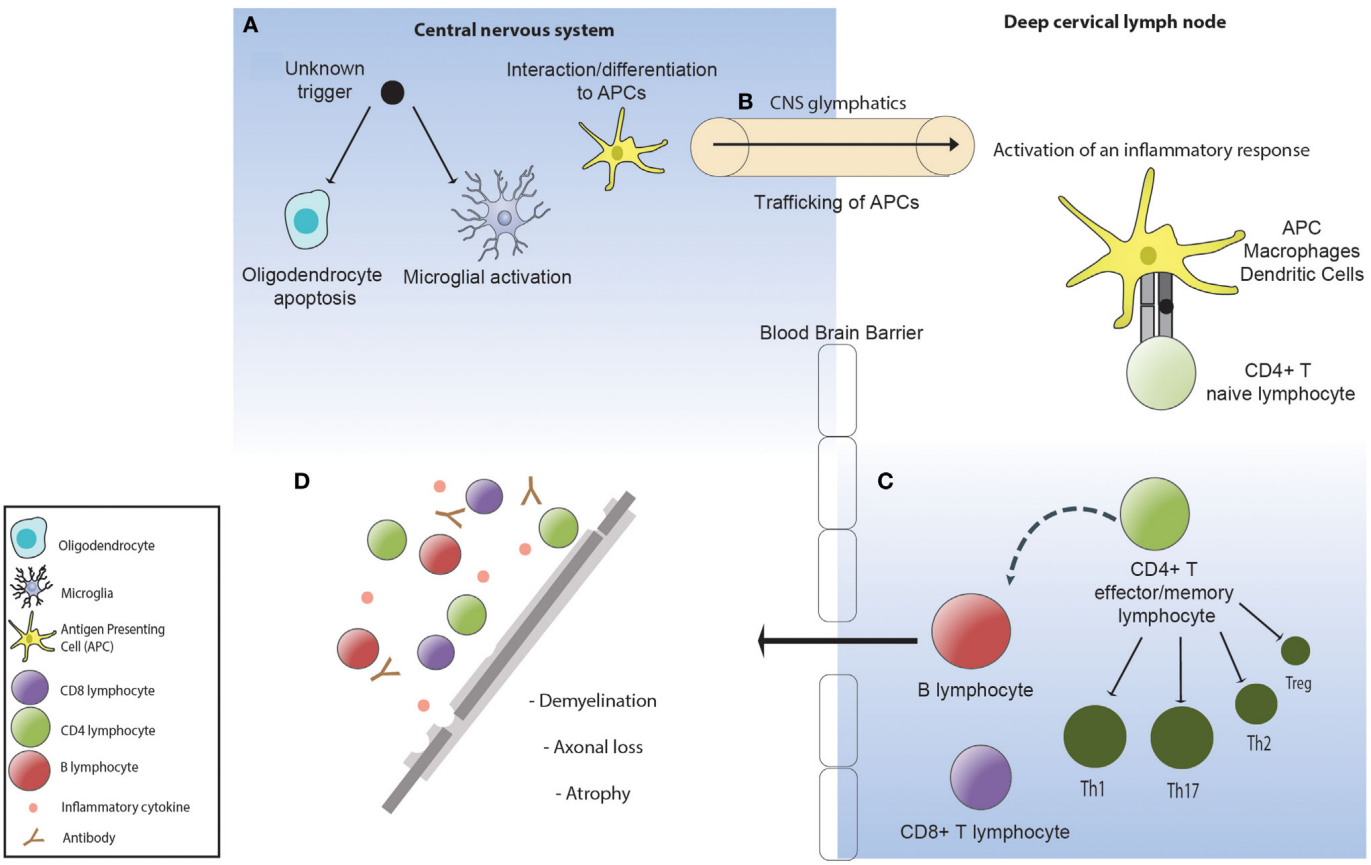
**(A)** An unknown factor triggers oligodendrocyte apoptosis. Microglia are activated and phagocytose the apoptotic oligodendrocytes. **(B)** Phagocytic cells containing oligodendrocyte myelin breakdown products traffic to the deep cervical lymph nodes *via* the central nervous system (CNS) lymphatics (glymphatics) where they activate an inflammatory immune response directed toward the undefined antigenic target of disease. **(C)** The inflammatory response in multiple sclerosis is defined by a dominance of Th1 and Th17 lymphocytes, pro-inflammatory cytokines, and impaired suppressor activity of Tregs. **(D)** Activated lymphocytes re-enter the CNS where they become re-activated and recruit local and systemic immune populations resulting in demyelination and subsequent axonal loss.

Both EAE and therapeutic trials of natalizumab, a humanized IgG4 monoclonal antibody against a4 integrin that blocks T cell trafficking into the CNS, have clearly demonstrated that a dysregulated immune response can be highly damaging to the brain and spinal cord. Moreover, the brain itself has long been recognized as an immune privileged site and an immunologically tolerogenic environment, raising important questions as to how pathological immune responses are generated and sustained in MS. In this regard, critical neuropathological evidence (67–70) has demonstrated that the earliest pathological change in an MS lesion is oligodendrocyte apoptosis and microglial activation in the absence of lymphocytic infiltration, with secondary demyelination (71). The recent identification of long hypothesized efferent CNS lymphatic pathways (72) and seminal allogeneic transplant experiments in the 1940s (73) demonstrate that the tolerogenic environment in the brain can be overcome by peripheral activation of an immune response. It is therefore plausible that CNS injury in MS arises as the result of peripheral activation of an aberrant adaptive immune response to an undefined antigen associated with oligodendroctye apoptosis (Figure 1).

**Figure 2:**
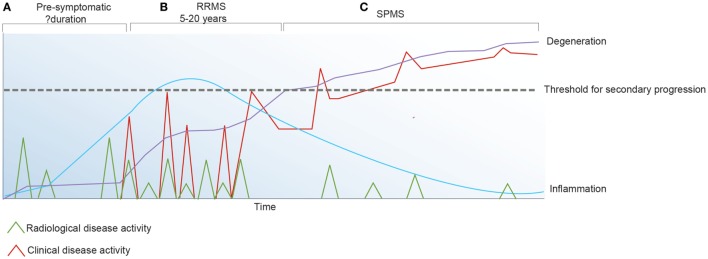
Inflammatory activity in multiple sclerosis (MS) may be detected clinically and/or radiologically. **(A)** The pre-symptomatic phase of the disease is defined by radiologically apparent relapses in the absence of clinical symptoms. **(B)** Following the first symptomatic demyelinating event, clinical and radiological relapses continue to occur. **(C)** Secondary progressive (SP) MS is defined by irreversible accumulation of disability due to chronic axonal loss which associates with ongoing brain atrophy and minimal inflammatory change on magnetic resonance imaging.

Although we are yet to define the antigenic target in MS or understand disease induction and how the immune system regulates the inflammatory changes that associate with the early relapsing-remitting disease course, it has been established that in addition to primary oligodendrocyte loss there is also marked axonal injury within the acute lesion (74). Over time, disability due to axonal injury accumulates and acute bouts of inflammation that associate with clinical relapses become less frequent (Figure 2). These observations favor the concept that IR therapies such as AHSCT should be applied early in the disease course when inflammatory changes are most prominent and prior to the accrual of irreversible neuroatrophy. In order to best understand the mechanisms underpinning IR therapies, it is essential to consider factors maintaining immune homeostasis in health and disease.

## The Lymphocyte “Steady State”

The circulating T lymphocyte pool is generated in early life *via* thymic development of T cells. Random and imprecise intra-thymic rearrangements of TCRA and TCRB genes generate rich TCR diversity (75) estimated to exceed 10^15^, with a circulating αßTCR repertoire in the range of 2.5 × 10^7^ (76). As thymocytes proliferate and mature into T cells, they undergo a series of distinct steps defined by changes in the expression of the TCR and the co-receptors, CD4 and CD8. T cells expressing the CD4 co-receptor are capable of interacting with MHC class II molecules present on antigen presenting cells, while CD8 expressing cells may be stimulated by any cell expressing MHC class I molecules. In health, approximately 50% of the circulating lymphocyte pool are T cells, with a dominance of CD4^+^ to CD8^+^ in a roughly 2:1 ratio. Naïve CD8 T cells emerging from the thymus are predestined to become cytotoxic cells, while CD4^+^ cells become “helper” lymphocytes whose fate is further determined during their first encounter with antigen. Mature naïve CD4^+^ or CD8^+^ cells survive in interphase for weeks to months in response to tonic TCR signals—weak, but significant stochastic interactions with self-peptide/MHC in the presence of IL-7 (77). Survival of these cells is determined by threshold “tuning,” which modulates the intensity of TCR signaling required for cell activation and proliferation (78).

Naïve T (TN) cells are defined conventionally by a host of receptors which facilitate lymphocyte entry to secondary lymph node organs, allowing these cells to interact with cognate antigen presented by APCs, resulting in conversion to a memory phenotype (78, 79). Activation of T lymphocytes enables splicing of pre-mRNA encoding CD45, a receptor-linked protein tyrosine phosphatase essential for TCR activation, resulting in expression of the prototypical antigen experienced T cell marker CD45RO (80). Antigen-specific proliferation is not only reliant on high affinity binding between MHC and the TCR, but co-stimulation in the context of IL-2, IL-4, IL-7, and IL-15 (79). In addition to conversion of phenotype from naïve to memory in response to antigen, a proportion of naïve cells undergo steady-state proliferation into memory-phenotype cells, in response to self-peptide, presumably with a lower degree of interaction than that required to trigger auto-inflammation (79). Following interaction with antigen, two major subsets of T memory (T_M_) cells develop (Table 1); those maintaining receptors enabling homing to lymphoid organs are known as central memory lymphocytes (T_CM_), with the alternate being effector T cells (T_E_) (79, 81–84). It has been further postulated that T_CM_ cells may differentiate into effector memory (T_EM_) or T_E_ cells on re-exposure to antigen (83, 85). More recently, a long-lived human memory T-cell population, termed T stem cell memory (T_SCM_) cells have been described (83). This population display functional attributes of naïve cells, with enhanced self-renewal and multipotent capacity to derive T_CM_, T_EM_, and T_E_ cells, in addition to memory characteristics including increased proliferative capacity and the ability to rapidly release cytokines on activation (82, 83). Phenotypically, this population express both traditional naïve and memory markers. It has been hypothesized that these T_SCM_ cells may be the earliest antigen experienced cells, and may serve as a reservoir of memory lymphocytes (82, 86). Costimulatory molecules CD27 and CD28 are present on memory cells; however, expression is lost as cells become terminally differentiated (82). In fact, terminally differentiated lymphocytes may even revert to a CD45RA phenotype, eventually transitioning to functional senescence or apoptosis (87, 88) defined by the expression of CD57 and CD58. Phenotypic, functional, and gene expression studies of T cell subsets suggest a non-linear continuum of lymphocyte development from T naïve to T stem cell memory, T central and effector memory, and finally terminal effector populations (83, 89), where less differentiated cells give rise to more differentiated progeny in response to antigenic or lymphopenic-induced proliferation requirements (79, 81–84).

**Table 1:** Model of T cell subsets by differentiation and phenotypical markers.

	T naïve	T stem cell memory	T central memory	T effector memory	T terminal effector
CD45RA	+	+	–	–	+
CD45RO	–	–	+	+	–
CCR7	+	+	+	–	–
CD62L	+	+	+	–	–
CD28	+	+	+	+/−	–
CD27	+	+	+	+/−	–
IL-7Rɑ	+	+	+	+/−	–
CD95	–	+	+	+	+
IL2Rβ	–	+	+	+	+
CD58	–	+	+	+	+
CD57	–	–	–	+/−	+

This model of “progressive differentiation” proposes that depending on the strength and quality of stimulatory signals, naïve lymphocytes are driven toward progressive stages of differentiation (78). More differentiated cells are under mounting feedback pressure to further differentiate or undergo apoptosis, culminating in the generation of terminally differentiated effector T cells which are prone to replicative senescence, reversing clonal population kinetics from growth to decline (87). Support for this model includes *ex vivo* analysis of virus-specific T cells, and *in vitro* differentiation studies (81, 90–92). In humans, progressive differentiation and replicative senescence has been associated with the generation of short lived T effector cells, expressing a CD8^+^CD28^−^CD57^+^ phenotype (93). These cells demonstrate reduced replicative potential, decreased telomere length, and a gene expression profile suggestive of a state of senescence (87, 88, 94), although immunosuppressive functions of this population have also been described (95). *In vitro* models have identified that CD4^+^ lymphocytes appear less prone to basal proliferation than CD8^+^ cells (96, 97), presumably relating to the ability of CD8^+^ lymphocytes to be activated by MHC class I molecules, present on all nucleated cells. Additionally, an elevated threshold for entering the cycle of progressive differentiation may serve as a mechanism to preserve the CD4^+^ population from senescence (98); however, the purpose of such variability remains unclear.

Interaction with cognate antigen and T cell activation results in ≈1,000 fold increase in size of the antigen-specific lymphocyte pool; however, it is estimated that less than 10% of cells activated after antigenic challenge survive in the memory pool (78) due to a process known as attrition (99), where a proportion of the expanded T cell pool are sacrificed to ensure “space” for newly developing T cells.

In addition to a retraction of clonal populations, response to antigen is also modulated over time. Studies in HIV, *M. tuberculosis* and Epstein–Barr virus (EBV) have informed our understanding of T cell anergy that occurs in the context of antigen persistence (100). Repeated antigen exposure has been shown to generate T cells that respond less intensely, producing lower levels of cytokine response than acutely stimulated effectors (101). Antigenic clearance is not required to stop clonal expansion of T_N_ cells. Instead, a protracted immune response can trigger a refractory state by adaptive “tuning” whereby ongoing antigenic stimulation may result in an activation-resistant state (78).

The ability to modulate response to antigen over time is a defining feature of adaptive immunity. Maintenance of immune homeostasis and prevention of auto-reactivity is mediated by regulatory T cells (Tregs), the primary drivers of peripheral tolerance due to their ability to suppress other cells in the immune system. The majority of Tregs are thymically derived (tTregs); cells that have been primed following their thymic interactions with self-peptide to constitutively express transcription factors (most notably FoxP3) that enable their suppressive function (102). Tregs may also differentiate from naïve conventional T cells under certain conditions, likely relating to TCR stimulation, cytokine environment, and associated epigenetic priming in the periphery (102, 103). Peripheral Tregs may be specific for both self and pathogen (103). Naïve Tregs are those which are yet to encounter cognate antigen in the periphery, while effector or activated Tregs have received strong antigenic stimulation evidenced by markers of proliferation and enhanced suppressive function (104). Activated Treg numbers fluctuate in the context of infection and appear to be modulated in order to thwart collateral damage to tissues exposed to chronic immune stimulation (105). Expanding literature reports a deficiency in the number and/or function of Tregs in autoimmune and inflammatory diseases, enabling a dysregulated immune response to antigen (106–111). This may reflect both a failure in induction of peripherally derived Tregs and inadequacies of thymic Treg production.

Thymic involution, an important aspect of immunosenescence, causes a decline in the output of conventional and regulatory naïve T cells from the thymus (112). It is therefore unsurprising that high-throughput TCR sequencing studies have shown that the TCRβ repertoire is most diverse in samples of umbilical cord blood and progressively decreases with age (113) so that in later life, the decline in naïve T cells and expansion of antigen experienced clones leads to a consistent decrease in TCR diversity (114). Furthermore, compensatory auto-proliferation in response to thymic involution has been postulated in the immunogenesis of inflammatory disorders (115–117). Molecular studies have confirmed that end-differentiated effector CD8 T cells, likely responsive to latent viruses such as cytomegalovirus (CMV), are expanded with advancing age, contributing to the decrease of TCR richness in memory, as opposed to naïve T cell pools (85, 118).

The natural history of MS is manifest by dissipation of CNS inflammation over time. Akin to the anergy that develops in response to chronic infection, it is possible that the later phase of MS, where inflammation is absent, relates to a “tuning” of the immune response. AHSCT, through the ablation of lymphocytes, induces IR bound by the principles of lymphocyte homeostasis outlined above. In addition to ablation of pathogenic clonal cells and the induction of thymopoiesis, it is proposed the AHSCT induces a recalibration of the immune system modifying the context in which antigen is first encountered and accelerating the induction of immunotolerance in the absence of relapse-associated disability.

## AHSCT in MS

In the context of MS, AHSCT encompasses a heterogeneous procedure involving five key stages: (i) mobilization of CD34^+^ hematopoietic stem cells (HSCs), (ii) HSC collection and preservation, (iii) immunoablative conditioning, (iv) HSC infusion, and (v) post-transplant care. Variations in AHSCT protocols exist between transplant units and no consensus exists regarding the optimal treatment regimen for MS (119). Mobilization of HSCs is undertaken by administering a marrow stimulating agent such as granulocyte colony-stimulating factor (G-CSF) and/or cyclophosphamide (Cyc) to induce proliferation and release of bone marrow HSCs, which are harvested from peripheral blood by leukoapheresis. Cyc is used not only to ablate pathogenic clones and increase HSC yield, but provides a degree of immunosuppression by lymphocyte depletion. In MS, Cyc is believed to counter the risk of exacerbating CNS inflammation that has been associated with G-CSF use (15). While a direct correlation exists between CD34^+^ cell dose and time to engraftment (120), *ex vivo* CD34^+^ selection of the leukoapheresis product is not only costly but has not been shown to improve outcomes in a recent retrospective analysis of autoimmune patients in the European Bone Marrow Transplantation (EBMT) database (119). One randomized trial of CD34^+^ selection in rheumatoid arthritis (121) and a recent retrospective study assessing CD34^+^ selection in systemic sclerosis (122) did not show added benefit to overall or progression-free survival. A 2013 publication showed a reduced relapse incidence in systemic lupus erythematosus (SLE) patients undergoing AHSCT with CD34^+^ selection (11 vs. 68%); however, a greater proportion of the non-graft selected patients had received a low-intensity conditioning regimen (123). Similarly, despite the complete arrest of MS disease activity post-AHSCT in the 2016 Atkins et al. trial (33), the use of a high-intensity myeloablative regimen makes it difficult to draw conclusions about the additional benefit of CD34^+^ selection. Therefore, although CD34^+^ selection is standard of care in some sites performing AHSCT for MS, compelling evidence to justify its use is lacking. Many centers opt instead for *in vivo* post-transplant T cell purging through the use of agents such as anti-thymocyte globulin (ATG) (119).

High-dose chemotherapy is used to ablate pathogenic lymphocytes, inducing pancytopenia and bone marrow aplasia prior to infusion of cryopreserved HSCs to variable intensity. Early clinical trials were designed on the back of animal studies (29, 30, 119, 124), suggesting a need for high-intensity immunoablation and employed myeloablative regimens using TBI and/or chemotherapeutics able to penetrate the blood–brain barrier. Higher intensity chemotherapy correlates with increasing adverse events, and a decreased tolerance for complications in the non-malignant setting has influenced treatment protocols in AIDs. Globally, the most widely used regimen continues to be BEAM—carmustine (BCNU), etoposide, cytarabine (AraC), and melphalan (15).

Treatment-related mortality (TRM) rates and MS disease progression following transplantation have limited the application of AHSCT in MS; however, historical results have been skewed by poor patient selection. An increase in the use of intermediate- and low-intensity regimens and better patient selection has resulted in a drop in the TRM reported by the EBMT. From 1995 to 2000, EBMT quoted a TRM rate of 7.3%, which fell to 1.3% in years 2001 to 2007 (125). However, in a 2017 meta-analysis of clinical trials for AHSCT in MS, TRM was 0.3% in the 349 patients who were transplanted after 2005 and no TRM was observed in those who underwent low-intensity conditioning (42). While determination of the optimal conditioning regimen in AHSCT for MS is of high research priority, a retrospective observational study from the EBMT autoimmune database (126) found that a transplant centers experience, and not intensity of conditioning had the strongest correlate with TRM.

The patient and treatment characteristics, results, and adverse events of pivotal trials of AHSCT in MS over the last 5 years have been summarized in Table 2 (33–37, 39–41, 127, 128) and recently reviewed elsewhere (18, 39, 120). A degree of disease stabilization has been seen in all trials of AHSCT in MS, and a multitude of recent publications support sustained disease suppression in a subset of patients with highly active inflammatory disease. A more profound treatment effect is observed with higher intensity conditioning (39), although it should be noted that methodological variability limits comparability between trials. It is notable that all studies show a durable response in the majority of RRMS patients and consistent relapse-free survival rates within this cohort, exceeding results seen in Phase 3 trials of alternate IR therapies (12, 13); however in the absence of randomized controlled trials, one must be cautious in drawing comparisons across treatments.

**Table 2:** Clinical and radiological outcomes in autologous hematopoietic stem cell transplantation (AHSCT) for MS.

Reference	Chen et al. (36)	Burman et al. (34)	Mancardi et al. (127)	Nash et al. (40)	Curro et al. (37)
Year	2012	2014	2015	2015—3 years/2017—5 years	2015

*N*# patients/gender	25/19 female	52/26 female (48 definite MS)	21 (9 AHSCT, 12 mitoxantrone)/14 female	24/17 female	7/3 female

Mean/median age (range)	37 (15–64)	31 (9–52)	36 (19–46)	37 (27–53)	28 (23–38)

Median baseline Expanded Disability Status Scale (EDSS) (range)	8.0 (3.0–9.5)	6.0 (1–8.5)	6.0 (5.5–6.5)	4.5 (3.0–5.5)	6 (5.0–7.0)

MS type	3 RR, 2 PR, 1 PP, 19 secondary progressive (SP)	40 RR, 1 PR, 2 PP, 5 SP	2 RR, 7 SP (AHSCT)	24 RR	7 RR

Median disease duration (months)	48 (7–147)	75 (4–300)	123 (24–276)	59 (7–144)	78 (48–144)

Mean/median follow-up (months)	59.6	47.4	48	46.5/62	range 36–60

Magnetic resonance imaging (MRI) activity at baseline	14 patients	66% MRI activity at baseline	ND	42%	100%

Conditioning regimen	Intermediate-intensity myeloablation (BEAM) + anti-thymocyte globulin (ATG)	Intermediate-intensity myeloablation (BEAM) + ATG, *n* = 41	Intermediate-intensity myeloablation (BEAM) + ATG	Intermediate-intensity myeloablation (BEAM) + ATG	Low-intensity lymphoablation Cyc + ATG
		Low-intensity lymphoablation Cyc + ATG, *n* = 7			

Outcome including relapse (event)-free survival (RFS)	PFS 74, 65, and 48% at 3, 6, and 9 years	4/48 relapses, ARR 0.03, all confirmed with new Gd^+^ MRI lesion	79% reduction in lesions compared to MTX arm	Event-free survival was 78.4% at 3 years based either on clinical or MRI findings	Single relapse in 1 patient at 3 years

	40% EDSS improvement, 28% EDSS stabilization	Median change in EDSS −0.75 (−7 to 1.5), −1.5 in relapsing-remitting multiple sclerosis (RRMS)	100% absence of Gd^+^ lesions during 4-year follow-up compared to 56% in MTX arm	Event-free survival was 69.2% at 5 years based either on clinical or MRI findings	6/7 had increase of EDSS at 3 years

	Follow-up MRI in 12 patients. 58% with Gd^+^ at baseline had no MRI activity on follow-up	5/48 new MRI activity	Reduced ARR as compared to MTX arm	RFS was 86.3% and PFS was 90.0% at 3 years	Mean Gd^+^/month decreased from 4.1 ± 4.1 pre-AHSCT to 0.3 ± 0.8 at 1 month and 0.2 ± 0.4 at 3 years

			No difference in EDSS or progression of disease	RFS was 86.9% and PFS was 91.3% at 5 years	6/7 developed Gd^+^ lesions after 5–6 months

Mortality <100 days	0	0	0	0	0

Mortality >100 days	1 pneumonia 4.5 months, 1 VZV hepatitis 15 months	0	0	1 MS progression <2.5 years, 1 asthma <3.5 years, 1 cardiac arrest 4.5 years	0

Adverse events/others	13 patients with bacteremia	0 malignancy, 1 Crohn’s Disease, 4 thyroid disease	3 severe AEs; 1 late engraftment, 1 systemic candidiasis/cytomegalovirus (CMV) reactivation, 1 ATG reaction		2/7 septicemia, 2/7 herpetic infections

Reference	Chen et al. (36)	Burman et al. (34)	Mancardi et al. (127)	Nash et al. (40)	Curro et al. (37)

	Burt et al. (35)	Shevchenko et al. (41)	Atkins et al. (33)	Cull et al. (128)	Muraro et al. (15, 39)

	2015	2015	2016	2017	2017

	151 (145 at end)/85 female	99/60 female	24 (21 at end)/14 female	13/11 female	281/164 female

	36 (18–60)	35 (18–55)	34 (24–45)	45 (22–60)	37 (15–65)

	4.0 (3.0–5.5)	3.5 (1.5–8.0)	5.0 (3.0–6.0)	7.0 (6.0–8.0)	6.0 (1.5–9.5)

	123 RR, 28 SP	43 RR, 3 PR, 18 PP, 35 SP	12 RR, 12 SP	3 PP, 10 SP	46 RR, 17 PR, 32 PP, 186 SP

	61 (9–264)	60 (6–288)	70 (16–134)	12.5 (36–360)	81 (<1–413)

	30	48.9	80.4	min. 36	79.2

	58%	40% MRI activity at baseline	87.5% 1 year pre-AHSCT	30.7% 1 year pre-AHSCT	N/A

	Low-intensity lymphoablation Cyc + ATG or alemtuzumab	Lesser than intermediate-intensity myeloablation (mini-BEAM like or carmustine + melphalan)	High-intensity myeloablation (busulfan + Cyc + ATG)	Low-intensity lymphoablation Cyc + ATG	Low intensity: Cyc ± ATG/alemtuzumab/Fludarabine
					Intermediate intensity: BEAM + ATG, BEAM, Cyc, TLI + melphalan, carmustine + Cyc + ATG High Intensity: Cyc + total body irradiation + ATG, busulfan + Cyc + ATG, busulfan + ATG

	RFS was 80% at 4 years	80% had event-free survival at median follow-up of 49 months	Event-free survival was 69.6% at 3 years	PFS was 69.6% at 3 years	PFS RR was 82% at 3 years, 73% at 5 years. PFS SP was 33% at 5 years

	PFS was 87% at 4 years	47% improved EDSS score by at least 0.5 points and 45% were stable after long-term follow-up	70% had no EDSS progression with a median follow-up of 6.7 years	0% EDSS improvement. 69% had no EDSS progression	OS 93% at 5 years and 84% at 10 years

	Mean Gd^+^ lesions 3.22 at 3–6 months pre-AHSCT to 0.08 at 5 years	MRI f/up in 55 patients. 15 patients had MRI activity at baseline, 3/15 developed Gd+ lesions on f/up	100% had absence of MRI activity after transplant	85% had absence of MRI activity after transplant	

	Decrease in T2 lesions volume from median of 8.57–5.74 cm^3^	Improved QoL in all RRMS at 1 year	35% had sustained improvement in EDSS		

	0	0	1 *Klebsiella* sepsis, 60 days	0	8 (2.8%)

	1 cardiovascular disease at 30 months	0	0	0	29

	1 bacteraemia, 4 zoster reactivation. 14% ITP with alemtuzumab, 3% ITP with ATG		Two patients censored within 2 years after CCSVI treatment	Febrile neutropenia 96%. 1 JCV cystitis, 1 CMV reactivation. 1 case ITP 12/12 post-AHSCT	New onset malignancy 9, autoimmune disease (AID) 14

## Immune Mechanisms of AHSCT in MS

**Figure 3:**
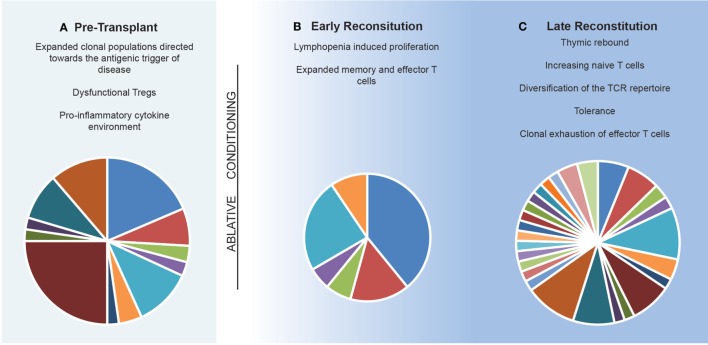
Model of the immune reconstitution occurring following autologous hematopoietic stem cell transplantation. **(A)** The pre-transplant environment is defined by clonally expanded populations of lymphocytes driving central nervous system inflammation with dysfunctional immunosuppressive lymphocytes. **(B)** Early reconstitution is driven by lymphopenia-induced proliferation of predominately CD8^+^ memory populations. Pie chart depicts T cell receptor (TCR) repertoire which is dominated by lymphopenia-induced proliferation induced expansions of clonal populations. **(C)** Late reconstitution involves a recovery of thymic output of naïve T cells. Pie chart depicts an increase in TCR diversity. Replicative senescence results in exhaustion of the previous expanded populations.

In general terms, IR therapies such as AHSCT are believed to exert their effects through (i) deletion of lymphocyte populations (129–133), (ii) induction of a lymphopenic state (129, 131, 134, 135), and (iii) subsequent development of a tolerant immune system which lacks the clonal expansion of functionally pathogenic T and B cells (131, 136) following recalibration of the TCR repertoire (Figure 3). This theoretical framework is supported by the observation that early clinical remission following AHSCT can be maintained for decades (33, 137). While the antigenic target of pathogenic lymphocytes is unique to each inflammatory disease, the sustained effects of AHSCT appear to relate less to MS-specific immune mechanisms, but more broadly to the principles of immune recalibration generated *via* the induction of lymphopenia and thymic reset. An exception to this is the ability of certain chemotherapeutics to cross the blood–brain barrier, enabling the elimination of MS-specific clones; however, this alone does not account for the durability of response observed in certain treatment trials. A recent meta-analysis and multi-center observational studies in MS have confirmed AHSCT to be a highly effective therapy, with event-free survival rates exceeding those seen best available pharmacotherapies (138). In order to optimize MS treatment in the future, it is vital to enhance our understanding of how IR therapies such as AHSCT adapt a patient’s immune response to the antigenic target of disease.

### Induction of the Lymphopenic State: Effect on Clonal Populations and Cell Phenotype

Ablative chemotherapy induces an extreme reduction in the mature lymphocyte pool and triggers potent proliferative signals for cells that have either survived the conditioning regimen or have been reinfused with the leukoapheresis product and survived subsequent *in vivo* T cell depletion (typically ATG). Studies of AHSCT in MS have confirmed a pattern of lymphocyte reconstitution akin to that seen in alloHSCT and AHSCT for hematological indications (128, 129, 131–133, 135), when similar conditioning regimens are used. However, the use of T cell-depleting agents post stem cell infusion is rare in the hematology setting (139–142). B cells, NK cells, and monocytes repopulate within the first weeks to 6 months (128, 131–133, 135) while CD3^+^ lymphocyte counts remain low in the initial post-transplant period, normalizing by 6–12 months (129, 133). Early proliferation of CD8^+^ T_M_ and T_E_ lymphocytes (131, 135) results in an inversion of the CD4^+^/CD8^+^ ratio out to 24 months (128, 129, 131, 132, 134, 135). This expansion of CD8 memory cells is termed “lymphopenia induced proliferation” (LIP), a process of activation and proliferation of leukocytes in a lymphopenic environment which serves as a response to rapidly restore T cell numbers. As in health, LIP promotes the expansion of CD8 more than CD4 lymphocytes (141). In theoretical terms, an “awareness” of available “space” (143) through alterations in the cytokine environment and availability of growth factors, induces lymphocytes to fill these voids. The mechanisms that result in LIP differ significantly from basal proliferation observed in the immune replete individual, whereby LIP is driven by a decreased threshold for TCR interactions with self-peptide MHC complexes and increased responsiveness to cytokines IL7, 15, and possibly 21 (144–146). This phenomenon has been observed in murine models in which rapid expansion of transferred lymphocytes occurs when injected to T cell deficient recipients, which is not seen on transfer to lymphoreplete mice (147).

Lymphopenia-induced proliferation results in lymphocyte phenotype switching, similar to those seen in the “progressive differentiation” model, with a rise in terminal effector populations. An early increase in the circulating CD8^+^CD28^−^CD57^+^ cells has been seen across studies of AHSCT in MS (129, 133, 135), and it has been suggested that this population is a significant contributor to the rise seen in the aforementioned CD8^+^ T_M_ population in the initial months post-AHSCT (133). These cells originate as an effector/cytotoxic population but may acquire an anergic, exhaustion-like, or regulatory phenotype (148–150), evidenced by weakened responses on TCR activation with lower cytokine release. Recent work in the field of systemic sclerosis demonstrated effector T cells undergo the greatest degree of expansion at 6 months post-AHSCT when compared with central or effector memory populations (151), and it is possible that the pace at which lymphocytes progress through cycles of differentiation and phenotype switching may be relevant to clinical outcomes. A study in type 1 diabetes mellitus demonstrated an early rise in T effector cells in the “short-response” population, opposed to the delayed rise in T central memory populations noted in the “prolonged-response” cohort (152). Recent work in MS has demonstrated that the inflammatory response of CD8^+^CD57^+^ cells correlates with relapse frequency, thereby confirming the relevance of this population to disease activity (153).

Molecular studies that have demonstrated a persistence of clonal populations following AHSCT despite patients remaining relapse free suggest that this early clonal expansion is dominated by lymphocytes reacting to common pathogens such as CMV or EBV (154, 155) and expansion of these terminal effector populations has been shown to predict viremia in the allogeneic transplant setting (156). However, the effect of these expansions on TCR diversity in the longer term remains unclear. Between 80 and 100% of MS patients are seropositive for EBV antibodies (157) and accumulating evidence demonstrates altered T-cell reactivity to EBV antigens (particularly EBNA) in MS (158). It has therefore been postulated that clonal expansions of EBV-specific T cells may shape the post-AHSCT TCR repertoire and limit the “space” available for diversification of the naïve T cell pool (133, 159–161). Notably, Muraro et al. identified an increase in the clonal frequency of EBV reactive T cells at 2 months in a subset of patients post-AHSCT and aforementioned investigations of CD8^+^CD57^+^ cells in MS demonstrates that a proportion of these cells are EBV specific (133). T cell competition ensures regulation of the size and diversity of the circulating T cell pool, confirmed by murine experiments of monoclonal TCR-transgenic cells demonstrating dramatically longer survival rates when seeded at low vs. high frequencies (162). While clonal competition between infused and residual cells post-AHSCT is likely to favor the broadening of the T cell repertoire, early sizeable expansions of clonotypes in response to common viruses may limit the available space for diversification of the lymphocyte pool post-transplant (160). However, clonal expansion can also lead to replicative senescence which is one potential explanation for attrition of pathogenic clones. The proportion of the mature T cell repertoire that needs to be eliminated for clinical efficacy in MS is unknown and hindered by the absence of a defined antigen. Although observational data supports the concept that a higher intensity conditioning regimen results in improved patient outcomes (42), it is not known whether this relates to deletion of pathogenic clonal populations or the degree and duration of lymphopenia. A high-throughput sequencing study in MS patients undergoing HSCT (161) has confirmed that the CD8^+^ lymphocyte pool is dominated by an expansion of pre-existing clones, while the CD4^+^ pool is primarily comprised of lymphocytes not present pre-transplant and increased TCR diversification trended toward patient response.

### Thymic Rebound and Diversification of the T Cell Pool

In contrast to the LIP of memory populations, a delayed repopulation of CD4^+^ T_N_ cells (128, 129, 131, 133–135), presumably thymic derived, occurs after 12–24 months and correlates with a rise in TCR excision circles (TREC) and CD31^+^ expression; a cell surface marker associated with recent thymic egress of CD4^+^ cells (128, 131, 133, 135). Reconstitution of naïve, thymic-derived T cells is postulated as the mechanism for increasing TCR repertoire diversity. This is supported by publications demonstrating a “Gaussian” repertoire following HSCT, typically 2–3 years post-transplant (133, 134, 140, 142, 163) and is consistent with the timing of thymic reactivation post-AHSCT. TREC levels are higher in AHSCT with CD34^+^ graft selection suggesting a correlation between CD34^+^ load and thymocyte development (164); however, a correlation with degree and duration of lymphopenia in graft selected cases cannot be excluded. Although maturation of T cells within the thymus declines with age (164–166), reactivation of the thymus plays a fundamental role in lymphocyte reconstitution after chemotherapy (133, 140, 163). Residual thymic tissue appears able to support thymopoiesis and generate TREC-bearing lymphocytes following AHSCT. Given the significance of thymic output in the durable diversification of the T cell repertoire following AHSCT, it is perhaps unsurprising that younger age, independent of disease duration, correlates with improved clinical outcomes in MS post-AHSCT (42).

### The Development of a Tolerant Environment

In ways still not fully understood, IR following AHSCT appears to favor the induction of tolerance. Both quantitative and qualitative abnormalities in Tregs have been observed in patients with inflammatory diseases including MS (167, 168) proposedly due to inadequate thymic production of naïve Tregs and impaired differentiation of memory Tregs (110, 111). Despite discrepancies in phenotypic definition, an early rise in Tregs post-AHSCT (129, 131, 132, 135) has been consistent across studies in MS. Early surges in Treg populations relative to the T cell pool are likely driven by LIP. Lymphopenia inducing therapies such as ATG and alemtuzumab have been independently associated with rapid expansions of CD4^+^CD25^+^ Tregs (169, 170), although the durability of these responses is unclear. Preliminary studies from our laboratory demonstrate an early spike in Tregs following AHSCT, which is not observed in lymphoma patients undergoing autografts with similar conditioning chemotherapy albeit without post-transplant ATG (171).

The sustained immune responses associated with AHSCT imply that a qualitative shift in the lymphocyte pool may relate to the context in which IR occurs. The significance of thymic reconstitution contributing to the restoration of Tregs has been highlighted by studies of recent thymic emigrants. Helios + Tregs, signaling thymic origin, have been shown to display greater proportions of RTE markers such as CD45RA and CD31^+^ in SLE patients following AHSCT, compared to those with active disease (172). Furthermore, a Dutch study looking at AHSCT in juvenile idiopathic arthritis demonstrated an oligoclonal Treg repertoire prior to AHSCT, which diversified post-transplant in all patients bar one, whose disease relapsed, implying a correlation between a diverse, presumably thymic-derived Treg repertoire and disease response (136). Tregs fell post-transplant in a cohort of SPMS patients (128) treated with a conditioning cyclophosphamide/ATG regimen but without post-transplant T cell depletion; however, it is unclear whether this difference relates to chemotherapeutics, patients age, disease stage, or a combination of the three.

Treg suppressive capability was shown to be decreased at baseline in systemic sclerosis patients undergoing AHSCT compared with controls, and normalized post-transplant (173). Furthermore, Burman et al. showed that Tregs remain capable of recognizing myelin oligodentrocyte glycoprotein post-AHSCT and are more capable of suppressing the associated Th17 response compared with MS patients on natalizumab (130). While further functional Treg experiments are, to our knowledge, yet to be performed in MS patients undergoing AHSCT, this is a point of interest for our laboratory.

Akin to the progressive differentiation of T cells, B cell development is defined by maturation of B lymphocytes with a transition of surface marker proteins defining phenotype and splicing of Ig heavy-chain transcripts to induce class-switching of surface Ig. B cells are believed to contribute to MS pathogenesis by means of antigen presentation, auto-antibody production, and cytokine regulation (174). Not only may oligoclonal B cell populations traffic between the CNS and peripheral circulation, but the formation of ectopic lymphoid aggregates within the meninges are purported to act as a germinal center, enabling a compartmentalized CNS site for activation and differentiation of T and B cells. Mounting evidence suggests antigen-dependent B cell subsets may have both pro-inflammatory and regulatory characteristics, with B regulatory cells (Bregs) emerging in different disease settings to exert a suppressive function (175). It is plausible that in addition to Treg dysfunction, inadequacies of Bregs may play a part in inflammatory disorders. Furthermore, a recent study of patients undergoing autografts for systemic sclerosis suggested that bone marrow “rebound” following AHSCT may induce proliferation of tolerant B cells (151). The reconstitution of these populations following IR therapy is yet to be studied in MS.

### An Altered Inflammatory Response

Phenotypic studies have confirmed reduced function of pro-inflammatory Th17 (IL-17A^+^IFN gamma^−^) (131) and Th1.17 (IL-17A^+^IFN gamma^+^) cells post-transplant (131, 132), supporting a functional correlate to the changes in regulatory T-cell networks (159). Furthermore, Darlington et al. demonstrated a reduction in IL-17A post-AHSCT (131), while staining for IFN gamma, TGF beta, IL-23 and CD8^+^ cytotoxic cytokine responses remained unchanged. By contrast, Sun et al. failed to find sustained difference in the cytokine profile post-AHSCT, despite persistent clinical responses (134). Despite an acknowledgment of the unidentified antigenic target(s) of MS, markers of autoreactivity have been frequently used a benchmark of the inflammatory state of disease. Studies analyzing changes in the populations of T cells targeting self-proteins such as myelin have persistently demonstrated spontaneous re-emergence of these populations following AHSCT (131, 134). While frequencies of autoreactive T cells do not appear effected by AHSCT, a reduced inflammatory Th17 response on MBP stimulation assays (131) supports the development of an altered antigenic response following AHSCT. A quantitative increase in circulating Tregs has associated with enhanced expression of immunoregulatory molecules such as CTLA-4 and glucocorticoid induced tumor necrosis factor-related protein, potentially modulating interactions between lymphocytes recruiting inflammatory cells to the CNS (176). Presumed pro-inflammatory mucosal-associated invariant T cells have been shown to be depleted post-AHSCT, to a greater degree than alternate IR therapies (135). Additionally, programmed cell death-1 protein (PD1), a cell surface marker known to downregulate inflammatory responses and induce tolerance following interaction with its ligand has been found to be more strongly expressed in lymphocytes following AHSCT, with degree of expression correlating with duration of disease remission (129, 132).

### Normalization of Immune Gene Expression

The importance of the environment in modulating gene expression is now well established, and it is likely that epigenetic changes induced by the lymphopenic state post-AHSCT may underlie many of the immunological modifications which occur following transplantation. Downregulation of microRNAs (miR-155, miR-142-3p, and miR-16) previously demonstrated to be upregulated in MS (176) and a concurrent increase in the expression of their normally silenced target genes (FOXP3, FOXO1, and IRF2BP2) has been demonstrated out to 2 years following AHSCT, all of which associate with maintenance of a tolerant cytokine environment. The downregulation of miR-16 (which targets PDCD1, the gene encoding PD-1) following AHSCT has been postulated to account for increased secretion of IL-10 following AHSCT (159). Gene expression profiles by microarray DNA-chip technology have been used to study CD4^+^ and CD8^+^ cells pre- and post-AHSCT (177), showing a normalization of differentially expressed genes (DEGs) toward that of healthy controls. Interestingly, CD8^+^ DEGs mirrored that of healthy controls at 2 years post-AHSCT, while CD4^+^ gene expression remained distinct from pre-transplant expression and that of controls. Work in the field of malignancy has suggested that the HSCT induced transcriptional changes correlate with the degree of myeloablation and forced bone marrow expansion (178), implying that the intensity of conditioning influences the pathogenicity of lymphocytes. While it is acknowledged that more profound therapeutic effects can be achieved with higher intensity conditioning (119), what remains unclear is whether this is simply a reflection of lymphopenia induced by the transplant and the subsequent effects of LIP with presumed replicative senescence of pathogenic clones and creation of space for TCR diversification, or whether there are chemotoxic effects that influence the biology of resident lymphoid progenitors in the bone marrow and thymus, resulting in enhanced proliferation following engraftment.

Defects of central and peripheral tolerance implicated in MS pathogenesis (179, 180) may also normalize post-AHSCT. Intrinsic pro-apoptotic genes, abnormal in MS patients at baseline when compared with healthy controls, normalized post-AHSCT. Interestingly these genetic modifications appeared more pronounced in patients treated with a more aggressive conditioning regimen. Genes regulating extrinsic apoptotic processes (FAS, FASL, and c-FLIPL) also corrected post-AHSCT (181), supporting a re-establishment of activation induced cell death and clonal tolerance.

### Unique Properties of AHSCT—Leukoapheresis Product

The potential for pathogenic clones within the graft product to be reinfused with CD34^+^ HSCs is a subject of ongoing debate. Theoretically, if the conditioning regimen is sufficiently intense to abrogate all lymphocytes from the body, the graft serves as the sole source for IR. It is therefore conceivable that graft manipulation would result in improved rates of remission. Despite this hypothesis (33), a randomized trial assessing the role of graft manipulation in AHSCT for Rheumatoid Arthritis conducted by our unit did not show clinical benefit (121). We postulate that this relates not only to increased adverse event rates outlined earlier, but the importance of infusion of Tregs with the graft product. Furthermore, the significance of clonal populations within the graft product remains unclear. A single study assessing TCR BV-BJ gene usage in 4 MS patients undergoing AHSCT was unable to identify shared clonotypes between the apheresis product and post-AHSCT peripheral blood, despite the presence of shared clones between the pre- and post-AHSCT peripheral blood specimens (154). Cyclophosphamide (Cyc) is a potent alkylating agent that induces DNA fragmentation, exerting its toxicity on mature lymphocytes, while CD34^+^ HSCs are relatively unaffected. While the leukoapheresis product is commonly evaluated pre-infusion with flow cytometry, the apoptotic potential of cells is not measured. Theoretically, cells entering the graft, which have been exposed to Cyc, would be less likely to engraft and proliferate, although recent work in the field of allogeneic transplant has suggested that a proportion of the engrafting cells are defined by a Cyc-resistant phenotype (182). The influence of ATG on these populations remains unknown and is under investigation within our laboratory.

## Alternate IR Therapies—Comparisons with AHSCT

Current therapeutic approaches in MS can be dichotomized to chronic immunosuppression, or IR therapy where pulsed induction treatments are used to rebuild the immune system. While the goal of IR therapies is to induce long-term disease remission, practical experience suggests that retreatment may be required. Given the increasing application of alemtuzumab, a humanized IgG3 monoclonal antibody which targets the CD52 receptor of lymphocytes, monocytes, and dendritic cells (13, 183), and the recent EMA and pending FDA approval for cladribine, a 2-chlorodeoxyadenosine triphosphate prodrug inhibiting lymphocyte proliferation (12), there is an urgent need for improved understanding of the effects these treatments have on the immune system in comparison with AHSCT. This has been outlined in Table 3 (12, 39, 42, 183–186) and explored further below.

**Table 3:** Comparison of IR therapies.

	Autologous hematopoietic stem cell transplantation (AHSCT)	Alemtuzumab	Cladribine
Mechanism of action	Lympho/myeloablative chemotherapeutic conditioning followed by autologous stem cell rescue	IgG3 mediated cell lysis of CD52 lymphocytes. Dosed at 0 and 12 months	Purine anti-proliferative oral therapy mediating selective lymphocyte apoptosis. Dosed at 0, 1, 12, and 13 months

Clinical efficacy (for RRMS)	Phase 1/2 trials and observational studies estimate MRI and clinical disease-free survival 78–83% at 2 years	Phase 3 treatment-controlled trials report MRI and clinical disease-free survival 39% at 2 years	Phase 3 placebo-controlled trials report 57.6% reduction in relapse rate, 74.4% relative reduction in combined unique lesions on MRI

CD4 repopulation	Memory cell counts approach baseline by 18–24 months	70–80% baseline at 12 and 24 months	Fall by 40–60%. Naïve cells fall to greater degree than memory cells

CD8 repopulation	Early reconstitution 3–6 months dominated by CD8 memory cells	Fall by 80–90% post-dosing, reach 50% baseline at 12 and 24 months	Fall by 20–40% from baseline after dosing

B cell repopulation	Approach baseline levels at 6–9 months	CD19^+^ cells return to baseline at 3–6 months, reach 120–130% prior to re-dosing	Fall by 90% after dosing, close to baseline prior to re-dosing at 12 months

Effect on thymic output	Multiple publications demonstrating increased CD4^+^CD31^+^ (RTE) and increased T cell receptor excision circle (TREC) post-AHSCT	Single study showing decrease in TREC post-alemtuzumab treatment	Unclear

Secondary AID	14/273 (5%) cases in largest longitudinal observational study	Estimated up to 50% at 7 years post treatment	None reported in Phase 3 trials

### Alemtuzumab

Alemtuzumab has been used historically for treatment of hematological malignancy and in organ and BMT and has recently been approved for the treatment of RRMS. Alemtuzumab use in MS patients is associated with improvement in disability, and a sustained reduction in both relapse rate and MRI disease activity for up to 5 years in 70% of patients (13, 183). However, IR following alemtuzumab treatment results in secondary AID in up to 50% of patients at 7 years post treatment (185). As such, use of alemtuzumab is typically reserved for patients with aggressive disease, akin to patients referred for AHSCT. Both treatments deplete T and B lymphocytes resulting in profound lymphopenia (184) followed by IR.

Following alemtuzumab treatment, T cell repopulation is driven by early homeostatic proliferation of predominately CD8^+^ memory lymphocytes (184) followed by slower reconstitution of CD4^+^ cells with median recovery time to baseline of 61 months following a single infusion (187). 47% of patients reached pre-treatment total lymphocyte counts by 12 months (188) with CD8^+^ populations approximating 50% of baseline and CD4^+^ cells reaching 30% of baseline at this time (187–190). In contrast to AHSCT, T_N_ cells do not approach pre-transplant percentages until 24 months (184, 190). The depleted CD4^+^ population is relatively enriched with CD4^+^CD25^+^ Tregs (188, 191), although absolute counts are significantly lower than baseline out to 24 months (184). Conversely, early B cell hyper-population is seen at 3–6 months post treatment correlating with a spike in B cell activating factor (BAFF) levels between 1 and 3 months (187, 189). The CD19^+^ population is dominated by an arrested phenotype of immature (CD19^+^CD27^−^CD38^+^CD10^+^) cells (180% of baseline counts at 3 months) and to a lesser degree mature naïve (CD19^+^CD27^−^CD38^+^CD10^−^) cells, while CD27^+^ memory B cell populations remain below 20% of baseline at 12 months (189). Thus, it is proposed that hyper-population of naïve B cells regenerating in the absence of Tregs may allow the development of autoreactive lymphocyte populations (184, 189). Elevated IL-21 levels, increased availability of antigen-presenting cells, and a lower threshold for cell proliferation in response to self-antigen skew the T cell repertoire following alemtuzumab therapy (192), further contributing to the development of secondary AID. Genetic studies have supported the role of IL-7 receptor polymorphisms in effecting CD4^+^ reconstitution after anti-retroviral therapy in HIV and lower rates of GVHD following allogeneic HSCT (193). Although IL7 receptor expression does not appear to affect autoimmunity (188), it is possible that in the lymphopenic environment, the MS associated IL-7R SNPs (194) may support the proliferation of CD4 and 8 cells to variable degrees and influence rates of secondary AID. Circulating CD34^+^ cells are present to a substantially lower extent in the post-alemtuzumab environment when compared to AHSCT, potentially explaining the prolonged lymphopenia following anti-CD52 therapy. Interestingly, TREC levels do not appear to be increased in alemtuzumab therapy (192), supporting the theory that higher CD34^+^ load more readily induces thymic T cell production. To determine whether these clear differences in mechanism of action between AHSCT and alemtuzumab correlate with differences in therapeutic efficacy will require a head-to-head trial.

### Cladribine

Cladribine is a purine analog whose active metabolite, 2-chlorodeoxyadenosine (2-cDK), is selectively accumulated in lymphocytes as a result of intracellular enzyme characteristics. 2-dCK exerts a lymphoablative effect *via* disruption of cellular metabolism and impairment of DNA synthesis. A 2010 phase III, placebo-controlled trial demonstrated a significantly lower annualized relapse rate compared with placebo, a higher relapse-free rate (79.7 vs. 60.9%) and a significant reduction in new brain lesions on MRI (12). Although initial clinical application was limited by concerns regarding infection and malignancy risk, it is anticipated that cladribine use will increase in the near future following recent studies that have led to a revised risk assessment (195).

T and B cells undergo a dose dependent depletion following cladribine induction (186). B cells drop to roughly 10% of baseline, reaching ≈80% of baseline prior to re-dosing. Reconstitution kinetics of B cells were notably different to the early hyper-population seen within alemtuzumab cohorts (186). CD4^+^ and CD8^+^ T cells undergo a similar pattern of depletion; with more marked suppression seen in the CD4^+^ population, reaching a nadir of 70% below baseline at 3 months in patients dosed at 5.25 mg/kg, and a 45% fall in the 3.5 mg/kg (EMA approved dose) cohort. CD8^+^ populations fell by 30–50% (186). Consistent with other IR therapies; T_N_ cells fell to a greater extent than T_M_ populations and reconstituted more slowly. CD4^+^ T_N_ cells fell by 80% 12 weeks after treatment cycle 1 and never rose above 70% of baseline. CD4^+^CD45RO^+^ T_M_ cells followed a similar pattern although recovered to 60–65%. CD8^+^ T_N_ cells fell to a lesser extent; roughly 25% at 12 weeks and the CD8^+^ T_M_ population only dropped by 10% (186). Although phase IV studies of cladribine are eagerly awaited, rates of secondary AID appear significantly lower than alemtuzumab (12). In regard to reconstitution profile, cladribine induction therapy mirrors the synchronized lymphocyte repopulation of AHSCT, reducing the risk of secondary autoimmunity. However, long-term clinical follow-up will be best placed to advise on the durability of this lympho-depleting therapy and future studies of deep immune phenotyping may offer further insights into the functional changes occurring in comparison with autologous transplantation.

## AHSCT: A “Fast Forward” of the Immune System

It is clear that a sustained, qualitative change in the immune system occurs after AHSCT. As outlined above, the T cell population in “health” reflects a balance of space, attrition, and replicative senescence. Akin to longitudinal studies of aging which suggest that a senescent phenotype of memory lymphocytes accumulate through progressive differentiation and chronic antigen exposure, a similar process clonal exhaustion may explain the transition from relapsing remitting to secondary progressive (SP) MS, where the inflammatory response to the aforementioned driver of oligodendrocyte apoptosis is abrogated over time.

In that vein, AHSCT may provide a mechanism of “accelerating” the immune response to antigen, inducing senescence and possibly apoptosis of pathogenic T cell populations as well as boosting mediators of immune tolerance, in the absence of relapse-associated disability. We postulate that this centers around the induction of significant lymphopenia triggering fundamental shifts in the circulating lymphocyte pool. The early ablation of a proportion of clonally expanded, antigen-specific lymphocytes through the use of cyclophosphamide in the mobilization regimen, the conditioning chemotherapy and *in* or *ex vivo* T cell depletion not only significantly decreases the load of pathogenic cells surviving AHSCT, but induces LIP. LIP drives cells through progressive phenotype switching, resulting in an early expansion of CD8^+^CD28^−^CD57^+^ terminally differentiated, effector lymphocytes. While the implication of this effector memory population remains debated, with some reviews suggesting an immunosuppressive effect on CD8^+^ cells (135), we favor a role equivalent to that defined in studies of antigen experienced lymphocytes (93) where a senescent phenotype is dominant. It is proposed that a proportion of these cells may be implicated in MS pathogenesis and LIP drives them to clonal exhaustion, rendering them no longer pathogenic.

Attrition describes the contraction of clonal lymphocytes that occurs as a result of competition for space between memory cells (196). Despite controversies regarding the required purity of the CD34^+^ leukoapheresis product, it is accepted that a proportion of circulating lymphocytes post-AHSCT have either survived the conditioning chemotherapy or post infusion serotherapy. Early LIP is likely to be dominated by surviving clones reacting to common pathogens such as CMV or EBV (156). Expansion of these populations may impact clonal space, and preliminary work in our laboratory has suggested that the degree of early CD8^+^ oligoclonality post-AHSCT correlates with CMV status. Through immunoablation and subsequent clonal exhaustion, a proportion of the expanded T cell pool are sacrificed, creating “space” to ensure the survival of naive lymphocytes post-AHSCT. Moreover, evidence exists to support the concept that these cells are thymic derived (131, 133, 164), which appears critical in the maintenance of a diverse T cell repertoire. Whether signaling between the thymus and peripheral drivers of proliferation influences kinetics of lymphocyte expansion remains unclear, although it has been proposed that RTEs may suppress expansion of peripheral memory T cells (160). It is clear that lymphopenia induces thymic rebound beyond the levels expected for age (140). Whether this thymic rebound is triggered by the degree of lymphopenia induced by the conditioning regimen, enhanced by the presence of HSCs which may demonstrate a superior ability to track to and engraft the thymus, or a combination of the two mechanisms, remains uncertain. Comparative studies with alemtuzumab suggest the importance of HSCs and RTEs in supporting synchronized T and B cell maturation (192). Lymphopenia not only promotes development of Treg populations but results in a series of epigenetic changes that may ensure durability of the tolerant phenotype after recovery of lymphocyte counts (129, 181). Following AHSCT, suppression of MS activity by clinical and radiologic measures appears to be accompanied by the reappearance of functional thymic T cell development, including Treg populations, enabling restoration of efficient antigen presentation.

Despite being highly efficacious in suppressing CNS inflammation and inducing disease stability IR therapies continue to be limited by morbidity and a small but significant mortality rate. There is evidence that IR therapies exert substantial changes in the quality of the immune system, indeed these changes are more likely to explain the induction of prolonged disease remission than the immunosuppression itself. Analysis of shifts in T cell populations over the period of IR allows the opportunity to gain significant insights into the pathogenesis of disease and treatment complications. Since the nucleotide sequence of the TCR can be used as a “fingerprint” of clonal populations, high-throughput sequencing analysis of individual T cell clones following IR therapy has the potential to monitor for pathogenic T cells. Development of targeted therapy in MS is however limited by our lack of identification of the antigenic target of disease. By identifying clonal T cell populations that correspond with disease (or an absence thereof) we envisage the potential for TCRs as a biomarker of MS, with utility in diagnosis, monitoring of treatment response or complications (AID) or for targeted cellular therapies in the future.

## Future Trials

Despite the increasing development of biologic therapies in MS, unmet needs exist for patients with aggressive and treatment-refractory MS. It is these patients who are at greatest risk of failing to respond to currently available disease-modifying therapy and in whom application of AHSCT is most appropriately targeted. More than 1,000 MS patients have been treated since 1997 and reported to the EBMT registry up until June 2017 (197). MS continues to be the most frequent indication for AHSCT in the non-malignant setting (197), with dominant countries of activity being Italy, Germany, Sweden, the United Kingdom, The Netherlands, Spain, France, and Australia. In the absence of a phase III, randomized controlled trial, autografts for MS should continue to be performed in the clinical trial setting and development of such trials is underway (18). In the United States, a National Institute of Health “BEAT-MS” trial will randomize patients to AHSCT or best available approved treatment including natalizumab, alemtuzumab, ocrelizumab, and potentially cladribine. The “NET-MS” study will follow a similar schema, while in Scandinavia the “RAM-MS” will compare AHSCT with alemtuzumab, with a similar “STAR-MS” study planned in the UK (18). It is hoped that these trials, along with the use of “real world” databases such as EBMT (197) and MSBase (198, 199) may better delineate which patients are most appropriately referred for AHSCT as opposed to alternate IR therapies, as this continues to be an undefined area of clinical practice influenced not only by MS severity but additional patient-specific factors.

Further investigation is also required to determine the optimal treatment protocol for AHSCT. Outstanding questions exist in regard to conditioning and mobilization regimens, which are unlikely to be answered in a head-to-head trial (15, 18). Attempts at modulating the “host environment” through the use of lymphodepletion or cytokine therapy has been trialed in the malignancy setting to improve engraftment and greater lymphocyte proliferation (200, 201). It has been postulated that establishing an anti-inflammatory environment prior to HSC infusion may benefit IR. Despite modest clinical benefit in Phase I–II clinical trials (202, 203), mesenchymal stem cells may possess unique immunomodulatory properties, inducing an anti-inflammatory milieu which may further enhance the benefits of AHSCT or other IR therapies (202–204).

## Conclusion

In order to improve therapeutic advances in MS, it is necessary to understand (i) what factors initiate the dysfunctional state, (ii) what are the antigenic target(s) of clonal T and B cell populations, and (iii) how a tolerant immune system is restored. Here, we have outlined crucial components of our hypothesis addressing these questions and summarized the role of AHSCT as an IR therapy. While many outstanding questions remain, we are developing a clearer understanding of the immunological shifts that are induced with AHSCT. It is hypothesized that accelerating the demise of pathogenic clones through LIP, replicative senescence and clonal attrition, the creation of “space” and the capacity for thymic rebound, regenerating a tolerant immune system from CD34^+^ HSCs are the core immunological shifts occurring in AHSCT. Future research identifying clonal T cell populations that correspond with disease and/or secondary autoimmunity and development of targeted T cell therapy remain the ultimate treatment goal. In the interim, exciting promise exists by closely examining these mechanisms in patients undergoing AHSCT.

## Author Contributions

JMassey conceptualized and designed the review, and drafted and edited the manuscript. IS conceptualized and designed the review and edited the manuscript. DM conceptualized the review and edited the manuscript. JMoore conceptualized the review and edited the manuscript.

## Conflict of Interest Statement

JMassey and IS have received honoraria for educational meetings from Biogen, Merck, and Genzyme. JMassey has received travel support to attend educational meetings from Biogen, Merck, and Genzyme. IS has served on Advisory Boards for Biogen and Merck. All other authors declare that the research was conducted in the absence of any commercial or financial relationships that could be construed as a potential conflict of interest.

## References

[1] Browne P Chandraratna D Angood C Tremlett H Baker C Taylor BV Atlas of multiple sclerosis 2013: a growing global problem with widespread inequity. Neurology (2014) 83(11):1022–4. 10.1212/WNL.0000000000000768 25200713 PMC4162299

[2] Eriksson M Andersen O Runmarker B. Long-term follow up of patients with clinically isolated syndromes, relapsing-remitting and secondary progressive multiple sclerosis. Mult Scler (2003) 9(3):260–74. 10.1191/1352458503ms914oa 12814173

[3] Tremlett H Paty D Devonshire V. Disability progression in multiple sclerosis is slower than previously reported. Neurology (2006) 66(2):172–7. 10.1212/01.wnl.0000194259.90286.fe 16434648

[4] Manouchehrinia A Tanasescu R Tench CR Constantinescu CS. Mortality in multiple sclerosis: meta-analysis of standardised mortality ratios. J Neurol Neurosurg Psychiatry (2016) 87(3):324–31. 10.1136/jnnp-2015-310361 25935887

[5] Paty DW Li DK. Interferon beta-1b is effective in relapsing-remitting multiple sclerosis. II. MRI analysis results of a multicenter, randomized, double-blind, placebo-controlled trial. UBC MS/MRI Study Group and the IFNB Multiple Sclerosis Study Group. Neurology (1993) 43(4):662–7. 10.1212/WNL.43.4.662 8469319

[6] Merkel B Butzkueven H Traboulsee AL Havrdova E Kalincik T. Timing of high-efficacy therapy in relapsing-remitting multiple sclerosis: a systematic review. Autoimmun Rev (2017) 16(6):658–65. 10.1016/j.autrev.2017.04.010 28428119

[7] Rush CAM MacLean HJ Freedman MS Aggressive multiple sclerosis: proposed definition and treatment algorithm. Nat Rev Neurol (2015) 11:379–89. 10.1038/nrneurol.2015.85 26032396

[8] Clifford DB De Luca A Simpson DM Arendt G Giovannoni G Nath A. Natalizumab-associated progressive multifocal leukoencephalopathy in patients with multiple sclerosis: lessons from 28 cases. Lancet Neurol (2010) 9(4):438–46. 10.1016/S1474-4422(10)70028-4 20298967

[9] Polman CH O’Connor PW Havrdova E Hutchinson M Kappos L Miller DH A randomized, placebo-controlled trial of natalizumab for relapsing multiple sclerosis. N Engl J Med (2006) 354(9):899–910. 10.1056/NEJMoa044397 16510744

[10] Calabresi PA Radue EW Goodin D Jeffery D Rammohan KW Reder AT Safety and efficacy of fingolimod in patients with relapsing-remitting multiple sclerosis (FREEDOMS II): a double-blind, randomised, placebo-controlled, phase 3 trial. Lancet Neurol (2014) 13(6):545–56. 10.1016/S1474-4422(14)70049-3 24685276

[11] Montalban X Hauser SL Kappos L Arnold DL Bar-Or A Comi G Ocrelizumab versus placebo in primary progressive multiple sclerosis. N Engl J Med (2017) 376:209–20. 10.1056/NEJMoa1606468 28002688

[12] Giovannoni G Comi G Cook S Rammohan K Rieckmann P Soelberg Sorensen P A placebo-controlled trial of oral cladribine for relapsing multiple sclerosis. N Engl J Med (2010) 362(5):416–26. 10.1056/NEJMoa0902533 20089960

[13] Coles AJ Twyman CL Arnold DL Cohen JA Confavreux C Fox EJ Alemtuzumab for patients with relapsing multiple sclerosis after disease-modifying therapy: a randomised controlled phase 3 trial. Lancet (2012) 380(9856):1829–39. 10.1016/S0140-6736(12)61768 23122650

[14] Kalincik T Brown JWL Robertson N Willis M Scolding N Rice CM Treatment effectiveness of alemtuzumab compared with natalizumab, fingolimod, and interferon beta in relapsing-remitting multiple sclerosis: a cohort study. Lancet Neurol (2017) 16(4):271–81. 10.1016/S1474-4422(17)30007-8 28209331

[15] Muraro PA Martin R Mancardi GL Nicholas R Sormani MP Saccardi R. Autologous haematopoietic stem cell transplantation for treatment of multiple sclerosis. Nat Rev Neurol (2017) 13(7):391–405. 10.1038/nrneurol.2017.81 28621766

[16] Arruda LC Clave E Moins-Teisserenc H Douay C Farge D Toubert A. Resetting the immune response after autologous hematopoietic stem cell transplantation for autoimmune diseases. Curr Res Transl Med (2016) 64(2):107–13. 10.1016/j.retram.2016.03.004 27316394

[17] Kelsey PJ Oliveira MC Badoglio M Sharrack B Farge D Snowden JA. Haematopoietic stem cell transplantation in autoimmune diseases: from basic science to clinical practice. Curr Res Transl Med (2016) 64(2):71–82. 10.1016/j.retram.2016.03.003 27316390

[18] Mancardi G Sormani MP Muraro PA Boffa G Saccardi R. Intense immunosuppression followed by autologous haematopoietic stem cell transplantation as a therapeutic strategy in aggressive forms of multiple sclerosis. Mult Scler (2007) 2007:1352458517742532. 10.1177/1352458517742532 29125439

[19] Swart JF Delemarre EM van Wijk F Boelens JJ Kuball J van Laar JM Haematopoietic stem cell transplantation for autoimmune diseases. Nat Rev Rheumatol (2017) 13(4):244–56. 10.1038/nrrheum.2017.7 28228650

[20] Ikehara S Good RA Nakamura T Sekita K Inoue S Oo MM Rationale for bone marrow transplantation in the treatment of autoimmune diseases. Proc Natl Acad Sci U S A (1985) 82(8):2483–7. 10.1073/pnas.82.8.2483 3887403 PMC397583

[21] Karussis DM Vourka-Karussis U Lehmann D Abramsky O Ben-Nun A Slavin S. Immunomodulation of autoimmunity in MRL/lpr mice with syngeneic bone marrow transplantation (SBMT). Clin Exp Immunol (1995) 100(1):111–7. 10.1111/j.1365-2249.1995.tb03611.x 7697909 PMC1534283

[22] Marmont AM Van Bekkum DW. Stem cell transplantation for severe autoimmune diseases: new proposals but still unanswered questions. Bone Marrow Transplant (1995) 16(4):497–8. 8528163

[23] Burt RK Burns W Ruvolo P Fischer A Shiao C Guimaraes A Syngeneic bone marrow transplantation eliminates V beta 8.2 T lymphocytes from the spinal cord of Lewis rats with experimental allergic encephalomyelitis. J Neurosci Res (1995) 41(4):526–31. 10.1002/jnr.490410412 7473884

[24] Burt RK Padilla J Begolka WS Canto MC Miller SD. Effect of disease stage on clinical outcome after syngeneic bone marrow transplantation for relapsing experimental autoimmune encephalomyelitis. Blood (1998) 91(7):2609–16. 9516163

[25] van Bekkum DW. Conditioning regimens for the treatment of experimental arthritis with autologous bone marrow transplantation. Bone Marrow Transplant (2000) 25(4):357–64. 10.1038/sj.bmt.1702153 10723577

[26] van Bekkum DW. Stem cell transplantation for autoimmune disorders. Preclinical experiments. Best Pract Res Clin Haematol (2004) 17(2):201–22. 10.1016/j.beha.2004.04.003 15302335

[27] Snowden JA Patton WN O’Donnell JL Hannah EE Hart DN. Prolonged remission of longstanding systemic lupus erythematosus after autologous bone marrow transplant for non-Hodgkin’s lymphoma. Bone Marrow Transplant (1997) 19(12):1247–50. 10.1038/sj.bmt.1700815 9208120

[28] Marmont AM Immunoablation followed or not by hematopoietic stem cells as an intense therapy for severe autoimmune diseases. New perspectives, new problems. Haematologica (2001) 86(4):337–45. 11325636

[29] van Gelder M van Bekkum DW. Treatment of relapsing experimental autoimmune encephalomyelitis in rats with allogeneic bone marrow transplantation from a resistant strain. Bone Marrow Transplant (1995) 16(3):343–51. 8535305

[30] van Gelder M Kinwel-Bohre EP van Bekkum DW. Treatment of experimental allergic encephalomyelitis in rats with total body irradiation and syngeneic BMT. Bone Marrow Transplant (1993) 11(3):233–41. 8467289

[31] van Gelder M van Bekkum DW. Effective treatment of relapsing experimental autoimmune encephalomyelitis with pseudoautologous bone marrow transplantation. Bone Marrow Transplant (1996) 18(6):1029–34. 8971369

[32] Fassas A Anagnostopoulos A Kazis A Kapinas K Sakellari I Kimiskidis V Peripheral blood stem cell transplantation in the treatment of progressive multiple sclerosis: first results of a pilot study. Bone Marrow Transplant (1997) 20(8):631–8. 10.1038/sj.bmt.1700944 9383225

[33] Atkins HL Bowman M Allan D Anstee G Arnold DL Bar-Or A Immunoablation and autologous haemopoietic stem-cell transplantation for aggressive multiple sclerosis: a multicentre single-group phase 2 trial. Lancet (2016) 388(10044):576–85. 10.1016/S0140-6736(16)30169-6 27291994

[34] Burman J Iacobaeus E Svenningsson A Lycke J Gunnarsson M Nilsson P Autologous haematopoietic stem cell transplantation for aggressive multiple sclerosis: the Swedish experience. J Neurol Neurosurg Psychiatry (2014) 85(10):1116–21. 10.1136/jnnp-2013-307207 24554104

[35] Burt RK Balabanov R Han X Sharrack B Morgan A Quigley K Association of nonmyeloablative hematopoietic stem cell transplantation with neurological disability in patients with relapsing-remitting multiple sclerosis. JAMA (2015) 313(3):275–84. 10.1001/jama.2014.17986 25602998

[36] Chen B Zhou M Ouyang J Zhou R Xu J Zhang Q Long-term efficacy of autologous haematopoietic stem cell transplantation in multiple sclerosis at a single institution in China. Neurol Sci (2012) 33(4):881–6. 10.1007/s10072-011-0859-y 22160751

[37] Curro’ D Vuolo L Gualandi F Bacigalupo A Roccatagliata L Capello E Low intensity lympho-ablative regimen followed by autologous hematopoietic stem cell transplantation in severe forms of multiple sclerosis: a MRI-based clinical study. Mult Scler (2015) 21(11):1423–30. 10.1177/1352458514564484 25583838

[38] Mancardi GL Sormani MP Di Gioia M Vuolo L Gualandi F Amato MP Autologous haematopoietic stem cell transplantation with an intermediate intensity conditioning regimen in multiple sclerosis: the Italian multi-centre experience. Mult Scler (2012) 18(6):835–42. 10.1177/1352458511429320 22127896

[39] Muraro PA Pasquini M Atkins HL Bowen JD Farge D Fassas A Long-term outcomes after autologous hematopoietic stem cell transplantation for multiple sclerosis. JAMA Neurol (2017) 74(4):459–69. 10.1001/jamaneurol.2016.5867 28241268 PMC5744858

[40] Nash RA Hutton GJ Racke MK Popat U Devine SM Griffith LM High-dose immunosuppressive therapy and autologous hematopoietic cell transplantation for relapsing-remitting multiple sclerosis (HALT-MS): a 3-year interim report. JAMA Neurol (2015) 72(2):159–69. 10.1001/jamaneurol.2014.3780 25546364 PMC5261862

[41] Shevchenko JL Kuznetsov AN Ionova TI Melnichenko VY Fedorenko DA Kurbatova KA Long-term outcomes of autologous hematopoietic stem cell transplantation with reduced-intensity conditioning in multiple sclerosis: physician’s and patient’s perspectives. Ann Hematol (2015) 94(7):1149–57. 10.1007/s00277-015-2337-8 25711670

[42] Sormani MP Muraro PA Schiavetti I Signori A Laroni A Saccardi R Autologous hematopoietic stem cell transplantation in multiple sclerosis: a meta-analysis. Neurology (2017) 88(22):2115–22. 10.1212/WNL.0000000000003987 28455383

[43] ClinicalTrials.gov Stem Cell Therapy for Patients with Multiple Sclerosis Failing Alternate Approved Therapy – A Randomized Study. NCT00273364. Chicago (2017).

[44] Jersild C Svejgaard A Fog T HL-A antigens and multiple sclerosis. Lancet (1972) 1(7762):1240–1. 10.1016/S0140-6736(72)90962-2 4113225

[45] Olerup O Hillert J. HLA class II-associated genetic susceptibility in multiple sclerosis: a critical evaluation. Tissue Antigens (1991) 38(1):1–15. 10.1111/j.1399-0039.1991.tb02029.x 1926129

[46] Sawcer S Hellenthal G Pirinen M Spencer CC Patsopoulos NA Moutsianas L Genetic risk and a primary role for cell-mediated immune mechanisms in multiple sclerosis. Nature (2011) 476(7359):214–9. 10.1038/nature10251 21833088 PMC3182531

[47] Schmidt H Williamson D Ashley-Koch A HLA-DR15 haplotype and multiple sclerosis: a HuGE review. Am J Epidemiol (2007) 165(10):1097–109. 10.1093/aje/kwk118 17329717

[48] Patsopoulos NA Esposito F Reischl J Lehr S Bauer D Heubach J Genome-wide meta-analysis identifies novel multiple sclerosis susceptibility loci. Ann Neurol (2011) 70(6):897–912. 10.1002/ana.22609 22190364 PMC3247076

[49] Parnell GP Booth DR The multiple sclerosis (MS) genetic risk factors indicate both acquired and innate immune cell subsets contribute to MS pathogenesis and identify novel therapeutic opportunities. Front Immunol (2017) 8:425 10.3389/fimmu.2017.00425 28458668 PMC5394466

[50] Salou M Garcia A Michel L Gainche-Salmon A Loussouarn D Nicol B Expanded CD8 T-cell sharing between periphery and CNS in multiple sclerosis. Ann Clin Transl Neurol (2015) 2(6):609–22. 10.1002/acn3.199 26125037 PMC4479522

[51] de Paula Alves Sousa A Johnson KR Nicholas R Darko S Price DA Douek DC Intrathecal T-cell clonal expansions in patients with multiple sclerosis. Ann Clin Trans Neurol (2016) 3(6):422–33. 10.1002/acn3.310 27547770 PMC4891996

[52] Link H Huang YM. Oligoclonal bands in multiple sclerosis cerebrospinal fluid: an update on methodology and clinical usefulness. J Neuroimmunol (2006) 180(1–2):17–28. 10.1016/j.jneuroim.2006.07.006 16945427

[53] Junker AI Ivanidze J Malotka J Eiglmeier I Lassmann H Wekerle H Multiple sclerosis: T-cell receptor expression in distinct brain regions. Brain (2007) 130:2789–99. 10.1093/brain/awm214 17890278

[54] Beecham AH Patsopoulos NA Xifara DK Davis MF Kemppinen A Cotsapas C Analysis of immune-related loci identifies 48 new susceptibility variants for multiple sclerosis. Nat Genet (2013) 45(11):1353–60. 10.1038/ng.2770 24076602 PMC3832895

[55] Dendrou CA Fugger L Friese MA. Immunopathology of multiple sclerosis. Nat Rev Immunol (2015) 15(9):545–58. 10.1038/nri3871 26250739

[56] Mein LE Hoch RM Dornmair K de Waal Malefyt R Bontrop RE Jonker M Encephalitogenic potential of myelin basic protein-specific T cells isolated from normal rhesus macaques. Am J Pathol (1997) 150(2):445–53. 9033260 PMC1858287

[57] Gold R Linington C Lassmann H. Understanding pathogenesis and therapy of multiple sclerosis via animal models: 70 years of merits and culprits in experimental autoimmune encephalomyelitis research. Brain (2006) 129(Pt 8):1953–71. 10.1093/brain/awl075 16632554

[58] Steinman L Zamvil SS. How to successfully apply animal studies in experimental allergic encephalomyelitis to research on multiple sclerosis. Ann Neurol (2006) 60(1):12–21. 10.1002/ana.20913 16802293

[59] Kitze B Pette M Rohrbach E Stadt D Kappos L Wekerle H Myelin-specific T lymphocytes in multiple sclerosis patients and healthy individuals. J Neuroimmunol (1988) 20(2–3):237 10.1016/0165-5728(88)90166-X 2461957

[60] Pette M Fujita K Kitze B Whitaker JN Albert E Kappos L Myelin basic protein-specific T lymphocyte lines from MS patients and healthy individuals. Neurology (1990) 40(11):1770–6. 10.1212/WNL.40.11.1770 1700336

[61] Ota K Matsui M Milford EL Mackin GA Weiner HL Hafler DA T-cell recognition of an immunodominant myelin basic protein epitope in multiple sclerosis. Nature (1990) 346(6280):183–7. 10.1038/346183a0 1694970

[62] Hunter SF Hafler DA Ubiquitous pathogens: links between infection and autoimmunity in MS? Neurology (2000) 55(2):164–5. 10.1212/WNL.55.2.164 10908883

[63] Chuong EB Elde NC Feschotte C. Regulatory evolution of innate immunity through co-option of endogenous retroviruses. Science (2016) 351(6277):1083–7. 10.1126/science.aad5497 26941318 PMC4887275

[64] Kremer D Forster M Schichel T Gottle P Hartung HP Perron H The neutralizing antibody GNbAC1 abrogates HERV-W envelope protein-mediated oligodendroglial maturation blockade. Mult Scler (2015) 21(9):1200–3. 10.1177/1352458514560926 25480862

[65] Alvord EC Jr Magee KR Kies MW Goldstein NP Clinico-pathologic correlations in experimental allergic encephalomyelitis. I. Observations on the early lesion. J Neuropathol Exp Neurol (1959) 18(3):442–6. 10.1097/00005072-195907000-00006 13665387

[66] Lucchinetti C Bruck W Parisi J Scheithauer B Rodriguez M Lassmann H. Heterogeneity of multiple sclerosis lesions: implications for the pathogenesis of demyelination. Ann Neurol (2000) 47(6):707–17. 10.1002/1531-8249(200006)47:6<707::AID-ANA3>3.0.CO;2-Q 10852536

[67] Barnett MH Prineas JW. Relapsing and remitting multiple sclerosis: pathology of the newly forming lesion. Ann Neurol (2004) 55(4):458–68. 10.1002/ana.20016 15048884

[68] Henderson AP Barnett MH Parratt JD Prineas JW. Multiple sclerosis: distribution of inflammatory cells in newly forming lesions. Ann Neurol (2009) 66(6):739–53. 10.1002/ana.21800 20035511

[69] Prineas JW Parratt JD. Oligodendrocytes and the early multiple sclerosis lesion. Ann Neurol (2012) 72(1):18–31. 10.1002/ana.23634 22829266

[70] Trapp BD Pathogenesis of multiple sclerosis: the eyes only see what the mind is prepared to comprehend. Ann Neurol (2004) 55(4):455–7. 10.1002/ana.20087 15048883

[71] Lassmann H Bradl M. Multiple sclerosis: experimental models and reality. Acta Neuropathol (2017) 133(2):223–44. 10.1007/s00401-016-1631-4 27766432 PMC5250666

[72] Iliff JJ Wang M Liao Y Plogg BA Peng W Gundersen GA A paravascular pathway facilitates CSF flow through the brain parenchyma and the clearance of interstitial solutes, including amyloid beta. Sci Trans Med (2012) 4(147):147ra11 10.1126/scitranslmed.3003748 PMC3551275 22896675

[73] Medawar PB Immunity to homologous grafted skin; the fate of skin homografts transplanted to the brain, to subcutaneous tissue, and to the anterior chamber of the eye. Br J Exp Pathol (1948) 29(1):58–69. 18865105 PMC2073079

[74] Trapp BD Peterson J Ransohoff RM Rudick R Mork S Bo L. Axonal transection in the lesions of multiple sclerosis. N Engl J Med (1998) 338(5):278–85. 10.1056/NEJM199801293380502 9445407

[75] Starr TK Jameson SC Hogquist KA. Positive and negative selection of T cells. Annu Rev Immunol (2003) 21:139–76. 10.1146/annurev.immunol.21.120601.141107 12414722

[76] Bonarius HP Baas F Remmerswaal EB van Lier RA ten Berge IJ Tak PP Monitoring the T-cell receptor repertoire at single-clone resolution. PLoS One (2006) 1:e55 10.1371/journal.pone.0000055 17183685 PMC1762342

[77] Sprent J Cho JH Boyman O Surh CD. T cell homeostasis. Immunol Cell Biol (2008) 86(4):312–9. 10.1038/icb.2008.12 18362947

[78] Grossman Z Min B Meier-Schellersheim M Paul WE Concomitant regulation of T-cell activation and homeostasis. Nat Rev Immunol (2004) 4(5):387–95. 10.1038/nri1355 15122204

[79] Sprent J Surh CD. Normal T cell homeostasis: the conversion of naive cells into memory-phenotype cells. Nat Immunol (2011) 12(6):478–84. 10.1038/ni.2018 21739670 PMC3434123

[80] Oberdoerffer S Moita LF Neems D Freitas RP Hacohen N Rao A. Regulation of CD45 alternative splicing by heterogeneous ribonucleoprotein, hnRNPLL. Science (2008) 321(5889):686–91. 10.1126/science.1157610 18669861 PMC2791692

[81] Sallusto F Geginat J Lanzavecchia A Central memory and effector memory T cell subsets: function, generation and maintenance. Annu Rev Immunol (2004) 22:745–63. 10.1146/annurev.immunol.22.012703.104702 15032595

[82] Gattinoni L Klebanoff CA Restifo NP. Paths to stemness: building the ultimate antitumour T cell. Nat Rev Cancer (2012) 12(10):671–84. 10.1038/nrc3322 22996603 PMC6352980

[83] Gattinoni L Lugli E Ji Y Pos Z Paulos CM Quigley MF A human memory T cell subset with stem cell-like properties. Nat Med (2011) 17(10):1290–7. 10.1038/nm.2446 21926977 PMC3192229

[84] Sallusto F Lenig D Forster R Lipp M Lanzavecchia A. Two subsets of memory T lymphocytes with distinct homing potentials and effector functions. Nature (1999) 401(6754):708–12. 10.1038/44385 10537110

[85] Newell EW Sigal N Bendall SC Nolan GP Davis MM. Cytometry by time-of-flight shows combinatorial cytokine expression and virus-specific cell niches within a continuum of CD8+ T cell phenotypes. Immunity (2012) 36(1):142–52. 10.1016/j.immuni.2012.01.002 22265676 PMC3752833

[86] Gattinoni L Speiser DE Lichterfeld M Bonini C T memory stem cells in health and disease. Nat Med (2017) 23(1):18–27. 10.1038/nm.4241 28060797 PMC6354775

[87] Effros RB Pawelec G. Replicative senescence of T cells: does the Hayflick Limit lead to immune exhaustion? Immunol Today (1997) 18(9):450–4. 10.1016/S0167-5699(97)01079-7 9293162

[88] Monteiro J Batliwalla F Ostrer H Gregersen PK. Shortened telomeres in clonally expanded CD28-CD8+ T cells imply a replicative history that is distinct from their CD28+CD8+ counterparts. J Immunol (1996) 156(10):3587–90. 8621891

[89] Mahnke YD Brodie TM Sallusto F Roederer M Lugli E. The who’s who of T-cell differentiation: human memory T-cell subsets. Eur J Immunol (2013) 43(11):2797–809. 10.1002/eji.201343751 24258910

[90] Holmes S He M Xu T Lee PP. Memory T cells have gene expression patterns intermediate between naive and effector. Proc Natl Acad Sci U S A (2005) 102(15):5519–23. 10.1073/pnas.0501437102 15809420 PMC556264

[91] Papagno L Spina CA Marchant A Salio M Rufer N Little S Immune activation and CD8+ T-cell differentiation towards senescence in HIV-1 infection. PLoS Biol (2004) 2(2):E20. 10.1371/journal.pbio.0020020 14966528 PMC340937

[92] Willinger T Freeman T Hasegawa H McMichael AJ Callan MF Molecular signatures distinguish human central memory from effector memory CD8 T cell subsets. J Immunol (2005) 175(9):5895–903. 10.4049/jimmunol.175.9.5895 16237082

[93] Fulop T Larbi A Pawelec G. Human T cell aging and the impact of persistent viral infections. Front Immunol (2013) 4:271. 10.3389/fimmu.2013.00271 24062739 PMC3772506

[94] Brenchley JM Karandikar NJ Betts MR Ambrozak DR Hill BJ Crotty LE Expression of CD57 defines replicative senescence and antigen-induced apoptotic death of CD8+ T cells. Blood (2003) 101(7):2711–20. 10.1182/blood-2002-07-2103 12433688

[95] Strioga M Pasukoniene V Characiejus D CD8(+) CD28(−) and CD8(+) CD57(+) T cells and their role in health and disease. Immunology (2011) 134(1):17–32. 10.1111/j.1365-2567.2011.03470.x 21711350 PMC3173691

[96] Cho JH Kim HO Surh CD Sprent J. T cell receptor-dependent regulation of lipid rafts controls naive CD8+ T cell homeostasis. Immunity (2010) 32(2):214–26. 10.1016/j.immuni.2009.11.014 20137986 PMC2830358

[97] Guimond M Veenstra RG Grindler DJ Zhang H Cui Y Murphy RD Interleukin 7 signaling in dendritic cells regulates the homeostatic proliferation and niche size of CD4+ T cells. Nat Immunol (2009) 10(2):149–57. 10.1038/ni.1695 19136960 PMC2713006

[98] Surh CD Sprent J Homeostasis of naive and memory T cells. Immunity (2008) 29(6):848–62. 10.1016/j.immuni.2008.11.002 19100699

[99] Jameson SC Maintaining the norm: T-cell homeostasis. Nat Rev Immunol (2002) 2(8):547–56. 10.1038/nri853 12154374

[100] Jelley-Gibbs DM Dibble JP Filipson S Haynes L Kemp RA Swain SL. Repeated stimulation of CD4 effector T cells can limit their protective function. J Exp Med (2005) 201(7):1101–12. 10.1084/jem.20041852 15795235 PMC2213138

[101] Martin MD Shan Q Xue HH Badovinac VP. Time and antigen-stimulation history influence memory CD8 T cell bystander responses. Front Immunol (2017) 8:634. 10.3389/fimmu.2017.00634 28642758 PMC5462920

[102] Ohkura N Kitagawa Y Sakaguchi S. Development and maintenance of regulatory T cells. Immunity (2013) 38(3):414–23. 10.1016/j.immuni.2013.03.002 23521883

[103] Rosenblum MD Way SS Abbas AK. Regulatory T cell memory. Nat Rev Immunol (2016) 16(2):90–101. 10.1038/nri.2015.1 26688349 PMC5113825

[104] Miyara M Gorochov G Ehrenstein M Musset L Sakaguchi S Amoura Z. Human FoxP3+ regulatory T cells in systemic autoimmune diseases. Autoimmun Rev (2011) 10(12):744–55. 10.1016/j.autrev.2011.05.004 21621000

[105] Burzyn D Benoist C Mathis D. Regulatory T cells in nonlymphoid tissues. Nat Immunol (2013) 14(10):1007–13. 10.1038/ni.2683 24048122 PMC4708287

[106] Banica L Besliu A Pistol G Stavaru C Ionescu R Forsea AM Quantification and molecular characterization of regulatory T cells in connective tissue diseases. Autoimmunity (2009) 42(1):41–9. 10.1080/08916930802282651 18800250

[107] Stalvey MS Brusko TM Mueller C Wasserfall CH Schatz DA Atkinson MA CFTR mutations impart elevated immune reactivity in a murine model of cystic fibrosis related diabetes. Cytokine (2008) 44(1):154–9. 10.1016/j.cyto.2008.07.468 18778952

[108] Bonelli M Savitskaya A von Dalwigk K Steiner CW Aletaha D Smolen JS Quantitative and qualitative deficiencies of regulatory T cells in patients with systemic lupus erythematosus (SLE). Int Immunol (2008) 20(7):861–8. 10.1093/intimm/dxn044 18469329

[109] Cao D Malmstrom V Baecher-Allan C Hafler D Klareskog L Trollmo C. Isolation and functional characterization of regulatory CD25brightCD4+ T cells from the target organ of patients with rheumatoid arthritis. Eur J Immunol (2003) 33(1):215–23. 10.1002/immu.200390024 12594850

[110] Viglietta V Baecher-Allan C Weiner HL Hafler DA Loss of functional suppression by CD4 CD25 regulatory T cells in patients with multiple sclerosis. J Exp Med (2004) 199(7):971–9. 10.1084/jem.20031579 15067033 PMC2211881

[111] Venken K Hellings N Broekmans T Hensen K Rummens JL Stinissen P. Natural naive CD4+CD25+CD127low regulatory T cell (Treg) development and function are disturbed in multiple sclerosis patients: recovery of memory Treg homeostasis during disease progression. J Immunol (2008) 180:6411–20. 10.4049/jimmunol.180.9.6411 18424765

[112] Linton PJ Dorshkind K. Age-related changes in lymphocyte development and function. Nat Immunol (2004) 5(2):133–9. 10.1038/ni1033 14749784

[113] Britanova OV Shugay M Merzlyak EM Staroverov DB Putintseva EV Turchaninova MA Dynamics of individual T cell repertoires: from cord blood to centenarians. J Immunol (2016) 196(12):5005–13. 10.4049/jimmunol.1600005 27183615

[114] Qi Q Liu Y Cheng Y Glanville J Zhang D Lee JY Diversity and clonal selection in the human T-cell repertoire. Proc Natl Acad Sci U S A (2014) 111(36):13139–44. 10.1073/pnas.1409155111 25157137 PMC4246948

[115] Krupica T Jr Fry TJ Mackall CL Autoimmunity during lymphopenia: a two-hit model. Clin Immunol (2006) 120(2):121–8. 10.1016/j.clim.2006.04.569 16766227

[116] Le Campion A Gagnerault MC Auffray C Becourt C Poitrasson-Riviere M Lallemand E Lymphopenia-induced spontaneous T-cell proliferation as a cofactor for autoimmune disease development. Blood (2009) 114(9):1784–93. 10.1182/blood-2008-12-192120 19561321

[117] Khoruts A Fraser JM A causal link between lymphopenia and autoimmunity. Immunol Lett (2005) 98(1):23–31. 10.1016/j.imlet.2004.10.022 15790505 PMC7126288

[118] Weekes MP Carmichael AJ Wills MR Mynard K Sissons JG. Human CD28-CD8+ T cells contain greatly expanded functional virus-specific memory CTL clones. J Immunol (1999) 162(12):7569–77. 10358214

[119] Snowden JA Saccardi R Allez M Ardizzone S Arnold R Cervera R Haematopoietic SCT in severe autoimmune diseases: updated guidelines of the European Group for Blood and Marrow Transplantation. Bone Marrow Transplant (2012) 47(6):770–90. 10.1038/bmt.2011.185 22002489 PMC3371413

[120] Curro DM Autologous hematopoietic stem cell transplantation in multiple sclerosis: 20 years of experience. Neurol Sci (2016) 37:857–65. 10.1007/s10072-016-2564-3 27071689

[121] Moore J Brooks P Milliken S Biggs J Ma D Handel M A pilot randomized trial comparing CD34-selected versus unmanipulated hemopoietic stem cell transplantation for severe, refractory rheumatoid arthritis. Arthritis Rheum (2002) 46(9):2301–9. 10.1002/art.10495 12355477

[122] Oliveira MC Labopin M Henes J Moore J Del Papa N Cras A Does ex vivo CD34+ positive selection influence outcome after autologous hematopoietic stem cell transplantation in systemic sclerosis patients? Bone Marrow Transplant (2016) 51(4):501–5. 10.1038/bmt.2015.299 26642332

[123] Alchi B Jayne D Labopin M Demin A Sergeevicheva V Alexander T Autologous haematopoietic stem cell transplantation for systemic lupus erythematosus: data from the European Group for Blood and Marrow Transplantation registry. Lupus (2013) 22(3):245–53. 10.1177/0961203312470729 23257404

[124] van Bekkum DW BMT in experimental autoimmune diseases. Bone Marrow Transplant (1993) 11(3):183–7. 8467280

[125] Saccardi R Kozak T Bocelli-Tyndall C Fassas A Kazis A Havrdova E Autologous stem cell transplantation for progressive multiple sclerosis: update of the European Group for Blood and Marrow Transplantation autoimmune diseases working party database. Mult Scler (2006) 12(6):814–23. 10.1177/1352458506071301 17263012

[126] Farge D Labopin M Tyndall A Fassas A Mancardi GL Van Laar J Autologous hematopoietic stem cell transplantation for autoimmune diseases: an observational study on 12 years’ experience from the European Group for Blood and Marrow Transplantation Working Party on Autoimmune Diseases. Haematologica (2010) 95(2):284–92. 10.3324/haematol.2009.013458 19773265 PMC2817032

[127] Mancardi GL Sormani MP Gualandi F Saiz A Carreras E Merelli E Autologous hematopoietic stem cell transplantation in multiple sclerosis A phase II trial. Neurology (2015) 84:981–8. 10.1212/WNL.0000000000001329 25672923

[128] Cull G Hall D Fabis-Pedrini M Carroll W Forster L Robins F Lymphocyte reconstitution following autologous stem cell transplantation for progressive MS. Mult Scler (2017) 3(1):2055217317700167. 10.1177/2055217317700167 28607754 PMC5415040

[129] Arruda LCM de Azevedo JTC de Oliveira GLV Scortegagna GT Rodrigues ES Palma PVB Immunological correlates of favorable long-term clinical outcome in multiple sclerosis patients after autologous hematopoietic stem cell transplantation. Clin Immunol (2016) 169:47–57. 10.1016/j.clim.2016.06.005 27318116

[130] Burman J Fransson M Tötterman TH Fagius J Mangsbo SM Loskog ASI T-cell responses after haematopoietic stem cell transplantation for aggressive relapsing–remitting multiple sclerosis. Immunology (2013) 140(2):211–9. 10.1111/imm.12129 23721329 PMC3784167

[131] Darlington PJ Touil T Doucet JS Gaucher D Zeidan J Gauchat D Diminished Th17 (not Th1) responses underlie multiple sclerosis disease abrogation after hematopoietic stem cell transplantation. Ann Neurol (2013) 73(3):341–54. 10.1002/ana.23784 23463494

[132] Karnell FG Lin D Motley S Duhen T Lim N Campbell DJ Reconstitution of immune cell populations in multiple sclerosis patients after autologous stem cell transplantation. Clin Exp Immunol (2017) 189(3):268–78. 10.1111/cei.12985 28498568 PMC5543487

[133] Muraro PA Douek DC Packer A Chung K Guenaga FJ Cassiani-Ingoni R Thymic output generates a new and diverse TCR repertoire after autologous stem cell transplantation in multiple sclerosis patients. J Exp Med (2005) 201(5):805–16. 10.1084/jem.20041679 15738052 PMC2212822

[134] Sun W Popat U Hutton G Zang YC Krance R Carrum G Characteristics of T-cell receptor repertoire and myelin-reactive T cells reconstituted from autologous haematopoietic stem-cell grafts in multiple sclerosis. Brain (2004) 127(Pt 5):996–1008. 10.1093/brain/awh117 14985264

[135] Abrahamsson SV Angelini DF Dubinsky AN Morel E Oh U Jones JL Non-myeloablative autologous haematopoietic stem cell transplantation expands regulatory cells and depletes IL-17 producing mucosal-associated invariant T cells in multiple sclerosis. Brain (2013) 136(Pt 9):2888–903. 10.1093/brain/awt182 23864273 PMC3754461

[136] Delemarre EM van den Broek T Mijnheer G Meerding J Wehrens EJ Olek S Autologous stem cell transplantation aids autoimmune patients by functional renewal and TCR diversification of regulatory T cells. Blood (2016) 127(1):91–101. 10.1182/blood-2015-06-649145 26480932

[137] Nash RA Hutton GJ Racke MK Popat U Devine SM Steinmiller KC High-dose immunosuppressive therapy and autologous HCT for relapsing-remitting MS. Neurology (2017) 88(9):842–52. 10.1212/WNL.0000000000003660 28148635 PMC5331868

[138] Sormani MP NEDA status in highly active MS can be more easily obtained with autologous hematopoietic stem cell transplantation than other drugs. Mult Scler (2016) 23(2):201–4. 10.1177/1352458516645670 27207454

[139] Mackall CL Bare CV Granger LA Sharrow SO Titus JA Gress RE. Thymic-independent T cell regeneration occurs via antigen-driven expansion of peripheral T cells resulting in a repertoire that is limited in diversity and prone to skewing. J Immunol (1996) 156(12):4609–16. 8648103

[140] Mackall CL Fleisher TA Brown MR Andrich MP Chen CC Feuerstein IM Age, thymopoiesis, and CD4+ T-lymphocyte regeneration after intensive chemotherapy. N Engl J Med (1995) 332(3):143–9. 10.1056/NEJM199501193320303 7800006

[141] Mackall CL Fleisher TA Brown MR Andrich MP Chen CC Feuerstein IM Distinctions between CD8+ and CD4+ T-cell regenerative pathways result in prolonged T-cell subset imbalance after intensive chemotherapy. Blood (1997) 89(10):3700–7. 9160675

[142] Peggs KS. Immune reconstitution following stem cell transplantation. Leuk Lymphoma (2004) 45(6):1093–101. 10.1080/10428190310001641260 15359987

[143] Hickman SP Turka LA Homeostatic T cell proliferation as a barrier to T cell tolerance. Philos Trans R Soc B Biol Sci (2005) 360(1461):1713–21. 10.1098/rstb.2005.1699 PMC1569537 16147536

[144] Tan JT Ernst B Kieper WC LeRoy E Sprent J Surh CD. Interleukin (IL)-15 and IL-7 jointly regulate homeostatic proliferation of memory phenotype CD8+ cells but are not required for memory phenotype CD4+ cells. J Exp Med (2002) 195(12):1523–32. 10.1084/jem.20020066 12070280 PMC2193564

[145] Dai Z Lakkis FG. Cutting edge: secondary lymphoid organs are essential for maintaining the CD4, but not CD8, naive T cell pool. J Immunol (2001) 167(12):6711–5. 10.4049/jimmunol.167.12.6711 11739484

[146] Ploix C Lo D Carson MJ A ligand for the chemokine receptor CCR7 can influence the homeostatic proliferation of CD4 T cells and progression of autoimmunity. J Immunol (2001) 167(12):6724–30. 10.4049/jimmunol.167.12.6724 11739486

[147] Surh CD Sprent J Homeostatic T cell proliferation: how far can T cells be activated to self-ligands? J Exp Med (2000) 192(4):F9–14. 10.1084/jem.192.4.F9 10952731 PMC2193242

[148] Gruener NH Lechner F Jung MC Diepolder H Gerlach T Lauer G Sustained dysfunction of antiviral CD8+ T lymphocytes after infection with hepatitis C virus. J Virol (2001) 75(12):5550–8. 10.1128/JVI.75.12.5550-5558.2001 11356962 PMC114267

[149] Kostense S Vandenberghe K Joling J Van Baarle D Nanlohy N Manting E Persistent numbers of tetramer+ CD8(+) T cells, but loss of interferon-gamma+ HIV-specific T cells during progression to AIDS. Blood (2002) 99(7):2505–11. 10.1182/blood.V99.7.2505 11895786

[150] Reignat S Webster GJ Brown D Ogg GS King A Seneviratne SL Escaping high viral load exhaustion: CD8 cells with altered tetramer binding in chronic hepatitis B virus infection. J Exp Med (2002) 195(9):1089–101. 10.1084/jem.20011723 11994415 PMC2193712

[151] Arruda LCM Malmegrim KCR Lima-Junior JR Clave E Dias JBE Moraes DA Immune rebound associates with a favorable clinical response to autologous HSCT in systemic sclerosis patients. Blood Adv (2018) 2(2):126–41. 10.1182/bloodadvances.2017011072 29365321 PMC5787873

[152] Malmegrim KC de Azevedo JT Arruda LC Abreu JR Couri CE de Oliveira GL Immunological balance is associated with clinical outcome after autologous hematopoietic stem cell transplantation in type 1 diabetes. Front Immunol (2017) 8:167. 10.3389/fimmu.2017.00167 28275376 PMC5319960

[153] Cencioni MT Magliozzi R Nicholas R Ali R Malik O Reynolds R Programmed death 1 is highly expressed on CD8+ CD57+ T cells in patients with stable multiple sclerosis and inhibits their cytotoxic response to Epstein-Barr virus. Immunology (2017) 152(4):660–76. 10.1111/imm.12808 28767147 PMC5680058

[154] Dubinsky AN Burt RK Martin R Muraro PA T-cell clones persisting in the circulation after autologous hematopoietic SCT are undetectable in the peripheral CD34+ selected graft. Bone Marrow Transplant (2010) 45(2):325–31. 10.1038/bmt.2009.139 19543329

[155] Storek J Zhao Z Liu Y Nash R McSweeney P Maloney DG Early recovery of CD4 T cell receptor diversity after “lymphoablative” conditioning and autologous CD34 cell transplantation. Biol Blood Marrow Transplant (2008) 14(12):1373–9. 10.1016/j.bbmt.2008.09.013 19041059 PMC2704065

[156] Suessmuth Y Mukherjee R Watkins B Koura DT Finstermeier K Desmarais C CMV reactivation drives posttransplant T-cell reconstitution and results in defects in the underlying TCRbeta repertoire. Blood (2015) 125(25):3835–50. 10.1182/blood-2015-03-631853 25852054 PMC4473113

[157] Disanto G Pakpoor J Morahan JM Hall C Meier UC Giovannoni G Epstein-Barr virus, latitude and multiple sclerosis. Mult Scler (2013) 19(3):362–5. 10.1177/1352458512451942 22767435

[158] Salvetti M Giovannoni G Aloisi F. Epstein-Barr virus and multiple sclerosis. Curr Opin Neurol (2009) 22(3):201–6. 10.1097/WCO.0b013e32832b4c8d 19359987

[159] Collins F Kazmi M Muraro PA. Progress and prospects for the use and the understanding of the mode of action of autologous hematopoietic stem cell transplantation in the treatment of multiple sclerosis. Expert Rev Clin Immunol (2017) 13(6):611–22. 10.1080/1744666X.2017.1297232 28277827

[160] Muraro PA Douek DC. Renewing the T cell repertoire to arrest autoimmune aggression. Trends Immunol (2006) 27(2):61–7. 10.1016/j.it.2005.12.003 16406806

[161] Muraro PA Robins H Malhotra S Howell M Phippard D Desmarais C T cell repertoire following autologous stem cell transplantation for multiple sclerosis. J Clin Invest (2014) 124(3):1168–72. 10.1172/JCI71691 24531550 PMC3934160

[162] Hataye J Moon JJ Khoruts A Reilly C Jenkins MK. Naive and memory CD4+ T cell survival controlled by clonal abundance. Science (2006) 312(5770):114–6. 10.1126/science.1124228 16513943

[163] Hakim FT Age-dependent incidence, time course, and consequences of thymic renewal in adults. J Clin Invest (2005) 115:930–9. 10.1172/JCI200522492 15776111 PMC1064981

[164] Douek DC Vescio RA Betts MR Brenchley JM Hill BJ Zhang L Assessment of thymic output in adults after haematopoietic stem-cell transplantation and prediction of T-cell reconstitution. Lancet (2000) 355(9218):1875–81. 10.1016/S0140-6736(00)02293-5 10866444

[165] Kohler ST Thiel A Life after the thymus: CD31 and CD31 human naive CD4 T-cell subsets. Blood (2009) 113(4):769–74. 10.1182/blood-2008-02-139154 18583570

[166] Douek DC McFarland RD Keiser PH Gage EA Massey JM Haynes BF Changes in thymic function with age and during the treatment of HIV infection. Nature (1998) 396(6712):690–5. 10.1038/25374 9872319

[167] Koetz K Bryl E Spickschen K O’Fallon WM Goronzy JJ Weyand CM T cell homeostasis in patients with rheumatoid arthritis. Proc Natl Acad Sci U S A (2000) 97(16):9203–8. 10.1073/pnas.97.16.9203 10922071 PMC16846

[168] Kayser C Alberto FL da Silva NP Andrade LE. Decreased number of T cells bearing TCR rearrangement excision circles (TREC) in active recent onset systemic lupus erythematosus. Lupus (2004) 13(12):906–11. 10.1191/0961203304lu2031oa 15645744

[169] De Mercanti S Alemtuzumab long-term immunologic effect: Treg suppressor function increases up to 24 months. Neurol Neuroimmunol Neuroinflamm (2016) 3(1):e194 10.1212/NXI.0000000000000194 26819963 PMC4723135

[170] Lopez M Clarkson MR Albin M Sayegh MH Najafian N. A novel mechanism of action for anti-thymocyte globulin: induction of CD4+CD25+Foxp3+ regulatory T cells. J Am Soc Nephrol (2006) 17(10):2844–53. 10.1681/ASN.2006050422 16914538

[171] Ford CDKM Zaunders J Hendrawan K Massey J Sutton I Milliken S, editors. Identification of the Immune Phenotypes Associated with Haematopoietic Stem Cell Transplantation for Multiple Sclerosis. Sydney, Australia: Haematology Society of Australia & New Zealand Congress (2017).

[172] Alexander T Sattler A Templin L Kohler S Gross C Meisel A Foxp3+ Helios+ regulatory T cells are expanded in active systemic lupus erythematosus. Ann Rheum Dis (2013) 72(9):1549–58. 10.1136/annrheumdis-2012-202216 23264341

[173] Baraut J Grigore EI Jean-Louis F Khelifa SH Durand C Verrecchia F Peripheral blood regulatory T cells in patients with diffuse systemic sclerosis (SSc) before and after autologous hematopoietic SCT: a pilot study. Bone Marrow Transplant (2014) 49(3):349–54. 10.1038/bmt.2013.202 24362364

[174] Jones AP Kermode AG Lucas RM Carroll WM Nolan D Hart PH. Circulating immune cells in multiple sclerosis. Clin Exp Immunol (2017) 187(2):193–203. 10.1111/cei.12878 27689339 PMC5217886

[175] Rosser EC Mauri C. Regulatory B cells: origin, phenotype, and function. Immunity (2015) 42(4):607–12. 10.1016/j.immuni.2015.04.005 25902480

[176] Arruda LLJ Sousa A Zanette D Palma P Panepucci R Brum D Autologous hematopoietic SCT normalizes miR-16, -155 and -142-3p expression in multiple sclerosis patients. Bone Marrow Transplant (2015) 50:380–9. 10.1038/bmt.2014.277 25486582

[177] de Paula A Sousa A Malmegrim KC Panepucci RA Brum DS Barreira AA Carlos Dos Santos A Autologous haematopoietic stem cell transplantation reduces abnormalities in the expression of immune genes in multiple sclerosis. Clin Sci (2015) 128:111–20. 10.1042/CS20140095 25116724

[178] Wood WA Krishnamurthy J Mitin N Torrice C Parker JS Snavely AC Chemotherapy and stem cell transplantation increase p16INK4a expression, a biomarker of T-cell aging. EBioMedicine (2016) 11:227–38. 10.1016/j.ebiom.2016.08.029 27591832 PMC5049997

[179] Gatzka M Walsh CM. Apoptotic signal transduction and T cell tolerance. Autoimmunity (2007) 40(6):442–52. 10.1080/08916930701464962 17729038

[180] Kinnunen T Chamberlain N Morbach H Cantaert T Lynch M Preston-Hurlburt P Specific peripheral B cell tolerance defects in patients with multiple sclerosis. J Clin Invest (2013) 123(6):2737–41. 10.1172/JCI68775 23676463 PMC3668812

[181] de Oliveira GL Ferreira AF Gasparotto EP Kashima S Covas DT Guerreiro CT Defective expression of apoptosis-related molecules in multiple sclerosis patients is normalized early after autologous haematopoietic stem cell transplantation. Clin Exp Immunol (2017) 187(3):383–98. 10.1111/cei.12895 28008595 PMC5290242

[182] Cieri NO Greco G Forcato R Taccioli M Cianciotti C Generation of human memory stem T cells after haploidentical T-replete hematopoietic stem cell transplantation. Blood (2015) 125:2865–74. 10.1182/blood-2014-11-608539 25736310

[183] Cohen JA Coles AJ Arnold DL Confavreux C Fox EJ Hartung HP Alemtuzumab versus interferon beta 1a as first-line treatment for patients with relapsing-remitting multiple sclerosis: a randomised controlled phase 3 trial. Lancet (2012) 380(9856):1819–28. 10.1016/S0140-6736(12)61769-3 23122652

[184] Baker D Herrod SS Alvarez-Gonzalez C Giovannoni G Schmierer K. Interpreting lymphocyte reconstitution data from the pivotal phase 3 trials of alemtuzumab. JAMA Neurol (2017) 74(8):961–9. 10.1001/jamaneurol.2017.0676 28604916 PMC5710323

[185] Tuohy O Costelloe L Hill-Cawthorne G Bjornson I Harding K Robertson N Alemtuzumab treatment of multiple sclerosis: long-term safety and efficacy. J Neurol Neurosurg Psychiatry (2015) 86(2):208–15. 10.1136/jnnp-2014-307721 24849515

[186] Baker D Herrod SS Alvarez-Gonzalez C Zalewski L Albor C Schmierer K. Both cladribine and alemtuzumab may effect MS via B-cell depletion. Neurol Neuroimmunol Neuroinflamm (2017) 4(4):e360. 10.1212/NXI.0000000000000360 28626781 PMC5459792

[187] Hill-Cawthorne GA Button T Tuohy O Jones JL May K Somerfield J Long term lymphocyte reconstitution after alemtuzumab treatment of multiple sclerosis. J Neurol Neurosurg Psychiatry (2012) 83(3):298–304. 10.1136/jnnp-2011-300826 22056965

[188] Cox AL Thompson SA Jones JL Robertson VH Hale G Waldmann H Lymphocyte homeostasis following therapeutic lymphocyte depletion in multiple sclerosis. Eur J Immunol (2005) 35(11):3332–42. 10.1002/eji.200535075 16231285

[189] Thompson SA Jones JL Cox AL Compston DA Coles AJ B-cell reconstitution and BAFF after alemtuzumab (Campath-1H) treatment of multiple sclerosis. J Clin Immunol (2010) 30(1):99–105. 10.1007/s10875-009-9327-3 19763798

[190] Zhang X Tao Y Chopra M Ahn M Marcus KL Choudhary N Differential reconstitution of T cell subsets following immunodepleting treatment with alemtuzumab (anti-CD52 monoclonal antibody) in patients with relapsing-remitting multiple sclerosis. J Immunol (2013) 191(12):5867–74. 10.4049/jimmunol.1301926 24198283

[191] Pant AB Wang Y Mielcarz DW Kasper EJ Telesford KM Mishra M Alteration of CD39+Foxp3+ CD4 T cell and cytokine levels in EAE/MS following anti-CD52 treatment. J Neuroimmunol (2017) 303:22–30. 10.1016/j.jneuroim.2016.12.010 28087077

[192] Jones JL Thompson SAJ Loh P Davies JL Tuohy OC Curry AJ Human autoimmunity after lymphocyte depletion is caused by homeostatic T-cell proliferation. Proc Natl Acad Sci U S A (2013) 110(50):20200–5. 10.1073/pnas.1313654110 24282306 PMC3864306

[193] Kielsen K Shamim Z Thiant S Faucher S Decker W Christensen IJ Soluble interleukin-7 receptor levels and risk of acute graft-versus-disease after allogeneic haematopoietic stem cell transplantation. Clin Immunol (2017). 10.1016/j.clim.2017.08.015 28863969

[194] McKay FC Swain LI Schibeci SD Rubio JP Kilpatrick TJ Heard RN Haplotypes of the interleukin 7 receptor alpha gene are correlated with altered expression in whole blood cells in multiple sclerosis. Genes Immun (2008) 9(1):1–6. 10.1038/sj.gene.6364436 17928869

[195] Pakpoor J Disanto G Altmann DR Pavitt S Turner BP Marta M No evidence for higher risk of cancer in patients with multiple sclerosis taking cladribine. Neurol Neuroimmunol Neuroinflamm (2015) 2(6):e158. 10.1212/NXI.0000000000000158 26468472 PMC4592538

[196] Takada K Jameson SC. Naive T cell homeostasis: from awareness of space to a sense of place. Nat Rev Immunol (2009) 9(12):823–32. 10.1038/nri2657 19935802

[197] Snowden JA Badoglio M Labopin M Giebel S McGrath E Marjanovic Z Evolution, trends, outcomes, and economics of hematopoietic stem cell transplantation in severe autoimmune diseases. Blood Adv (2017) 1(27):2742–55. 10.1182/bloodadvances.2017010041 29296926 PMC5745133

[198] Butzkueven H Chapman J Cristiano E Grand’Maison F Hoffmann M Izquierdo G MSBase: an international, online registry and platform for collaborative outcomes research in multiple sclerosis. Mult Scler (2006) 12(6):769–74. 10.1177/1352458506070775 17263005

[199] Kalincik T Kuhle J Pucci E Rojas JI Tsolaki M Sirbu CA Data quality evaluation for observational multiple sclerosis registries. Mult Scler (2017) 23(5):647–55. 10.1177/1352458516662728 27481209

[200] Miller JS Weisdorf DJ Burns LJ Slungaard A Wagner JE Verneris MR Lymphodepletion followed by donor lymphocyte infusion (DLI) causes significantly more acute graft-versus-host disease than DLI alone. Blood (2007) 110(7):2761–3. 10.1182/blood-2007-05-090340 17579184 PMC1988949

[201] Tanaka T Watanabe S Takahashi M Sato K Saida Y Baba J Transfer of in vitro-expanded naive T cells after lymphodepletion enhances antitumor immunity through the induction of polyclonal antitumor effector T cells. PLoS One (2017) 12(8):e0183976 10.1371/journal.pone.0183976 28854279 PMC5576657

[202] Connick P Kolappan M Crawley C Webber DJ Patani R Michell AW Autologous mesenchymal stem cells for the treatment of secondary progressive multiple sclerosis: an open-label phase 2a proof-of-concept study. Lancet Neurol (2012) 11(2):150–6. 10.1016/S1474-4422(11)70305-2 22236384 PMC3279697

[203] Llufriu S Sepulveda M Blanco Y Marin P Moreno B Berenguer J Randomized placebo-controlled phase II trial of autologous mesenchymal stem cells in multiple sclerosis. PLoS One (2014) 9(12):e113936. 10.1371/journal.pone.0113936 25436769 PMC4250058

[204] Harris VK Faroqui R Vyshkina T Sadiq SA. Characterization of autologous mesenchymal stem cell-derived neural progenitors as a feasible source of stem cells for central nervous system applications in multiple sclerosis. Stem Cells Transl Med (2012) 1(7):536–47. 10.5966/sctm.2012-0015 23197858 PMC3659719

